# Identifying Critical States of Complex Diseases by Single-Sample Jensen-Shannon Divergence

**DOI:** 10.3389/fonc.2021.684781

**Published:** 2021-06-04

**Authors:** Jinling Yan, Peiluan Li, Rong Gao, Ying Li, Luonan Chen

**Affiliations:** ^1^ School of Mathematics and Statistics, Henan University of Science and Technology, Luoyang, China; ^2^ State Key Laboratory of Cell Biology, Institute of Biochemistry and Cell Biology, Center for Excellence in Molecular Cell Science, Chinese Academy of Sciences, Shanghai, China; ^3^ Center for Excellence in Animal Evolution and Genetics, Chinese Academy of Sciences, Kunming, China; ^4^ Key Laboratory of Systems Health Science of Zhejiang Province, Hangzhou Institute for Advanced Study, University of Chinese Academy of Sciences, Hangzhou, China; ^5^ School of Life Science and Technology, ShanghaiTech University, Shanghai, China

**Keywords:** complex disease, critical state, sJSD signal biomarker, single-sample Jensen-Shannon divergence (sJSD), dynamic network biomarker (DNB), dark genes

## Abstract

**Motivation:**

The evolution of complex diseases can be modeled as a time-dependent nonlinear dynamic system, and its progression can be divided into three states, i.e., the normal state, the pre-disease state and the disease state. The sudden deterioration of the disease can be regarded as the state transition of the dynamic system at the critical state or pre-disease state. How to detect the critical state of an individual before the disease state based on single-sample data has attracted many researchers’ attention.

**Methods:**

In this study, we proposed a novel approach, i.e., single-sample-based Jensen-Shannon Divergence (sJSD) method to detect the early-warning signals of complex diseases before critical transitions based on individual single-sample data. The method aims to construct score index based on sJSD, namely, inconsistency index (ICI).

**Results:**

This method is applied to five real datasets, including prostate cancer, bladder urothelial carcinoma, influenza virus infection, cervical squamous cell carcinoma and endocervical adenocarcinoma and pancreatic adenocarcinoma. The critical states of 5 datasets with their corresponding sJSD signal biomarkers are successfully identified to diagnose and predict each individual sample, and some “dark genes” that without differential expressions but are sensitive to ICI score were revealed. This method is a data-driven and model-free method, which can be applied to not only disease prediction on individuals but also targeted drug design of each disease. At the same time, the identification of sJSD signal biomarkers is also of great significance for studying the molecular mechanism of disease progression from a dynamic perspective.

## Introduction

**Figure 1:**
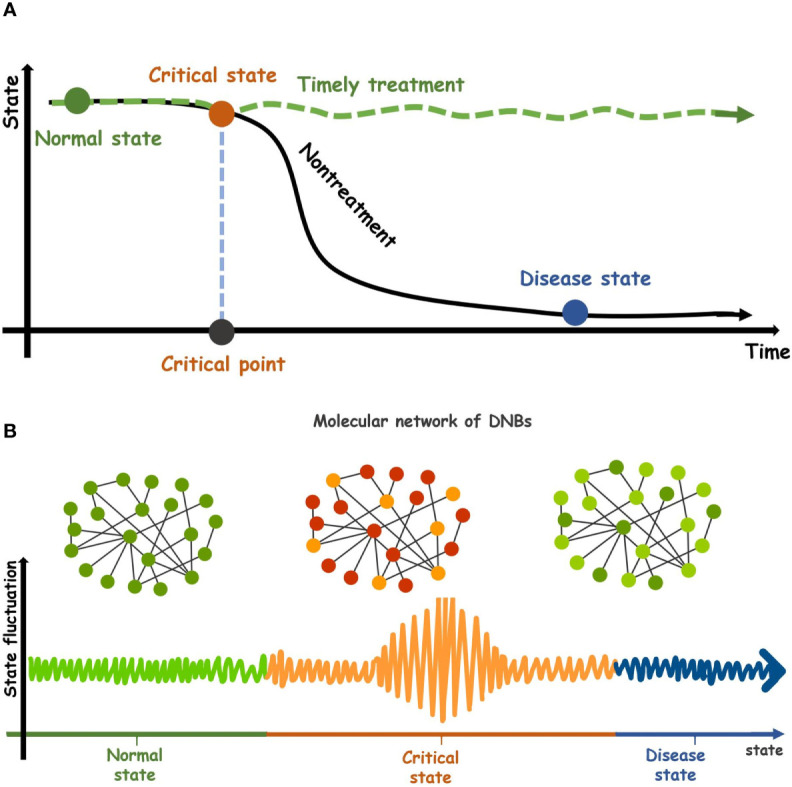
Dynamic evolution of complex diseases. **(A)** The dynamic characteristics of a complex disease. Its progression can be divided into three states, a relatively healthy state (normal state), a critical transition state (pre-disease state), and a disease state. After the critical state of complex diseases is successfully detected and given timely treatment, the system will return from the critical state to the normal stage (green curve). Conversely, the system will go through the critical stage and enter the disease state. (black curve). **(B)** Three states in the molecular networks of DNBs during disease progression. The critical state is the limit point of normal state and is characterized by low stability and robustness.

Complex diseases seriously endanger human health. They often occur as a result of multiple molecular interactions (1). In the progression of diseases, some develop relatively slowly and can usually be controlled by drug intervention and health care. However, many complex or chronic diseases undergo drastic or qualitative changes resulting from various internal or external factors (2). Take cancer, for example. Most cancers have no obvious symptoms in the early stage and are extremely difficult to cure when found in the late stage. Metabolic diseases are often irreversible. Therefore, early prevention and diagnosis of complex diseases are essential (3). The evolution of complex diseases can be modeled as a time-related nonlinear dynamic system, and the sudden deterioration of diseases can be regarded as the state transformation of the dynamic system at the tipping point in cases, for example, type-2 diabetes (4), colorectal tumors (5) and breast cancer (6). According to the dynamics of a complex disease, its progression can be divided into three states: a relatively healthy state (normal state), a critical state (pre-disease state), and a disease state (**Figure 1**). Under normal state, the biological system is stable and changes slowly, characterized by stability and robustness. Under the critical state or tipping point, the biological system is at the limit point of the normal state. If the system is disturbed from the outside, it is likely to enter the next stationary state, i.e., disease state, or return to the former stationary state, i.e., a normal state with high reversibility. The disease state indicates that the system has passed a critical state into a new stable state, and the disease is in a phase of deterioration, in which most patients develop symptoms of the disease and begin to receive treatment, but it is difficult to return to the normal state. Therefore, detecting the early warning signals by identifying the critical stage/pre-disease stage is crucial to prevent the catastrophic deterioration of complex diseases (7). There is no significant difference in symptoms between the pre-disease state and the normal state in the process of complex disease, so it is very difficult to detect the critical transition during disease progression through traditional molecular markers and network markers (8).

In order to further study the pre-disease state between the normal state and the disease stage, a new type of dynamic network biomarker (DNB) was proposed. DNBs are a set of biomolecules with strong dynamic correlation, and the molecular concentration presents dynamic changes rather than keeping the constant value of the critical state (7). DNB reveals early warning signals of critical transitions before the deterioration of complex diseases. The DNB method has been applied to some real disease datasets and identified the pre-disease states of several diseases such as the early warning signals for detecting type 1 diabetes and its main biomolecular network (9), identifying the differentiation status of breast cancer McF-7 cells (10), and detecting the early warning signals for influenza outbreak (11). The application of DNB has achieved good results. However, the DNB index’s construction depends on three statistical conditions, i.e., the standard deviation of DNB inter-molecules, the correlation coefficient of DNB internal molecules, and the correlation coefficient between the internal biomolecules and the external biomolecules of DNB. The calculation of these indicators requires a large number of samples, which is difficult to achieve for many biomedical research studies. Therefore, the need for multiple samples in the validation of real datasets distinctly limits DNB’s application.

**Figure 2:**
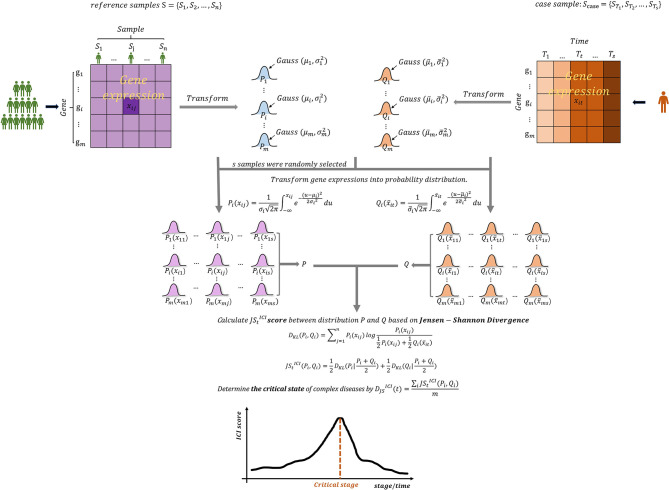
The outline for detecting early warning signal of pre-disease state based on sJSD. Given reference samples and case samples, the Gaussian distribution was fitted for each gene, and the probability distribution area was converted. The inconsistency index (ICI) which can be used to identify early-warning signals for deterioration of complex diseases are constructed based on the sJSD theory.

With the advances of bio-experimental technology, especially the widespread application of microarray chip technology, a large number of high-throughput biological data has been generated. These data and information contain the internal correlation between genes and life characteristics, providing an opportunity for further research and understanding of the pathogenesis and development of complex diseases (12). To further quantify state transitions in biological systems, the probability distributions have been introduced to study biomolecular observations. From the probability distribution perspective, the similarity and difference of two variables or indicators can be measured through the theory of Jensen-Shannon Divergence (JSD). This feature of JSD is of great significance for the detection of the pre-disease state of complex diseases. Motivated by this point, we develop an approach, the single-sample Jensen-Shannon Divergence (sJSD), which can quantify the information loss when the reference distribution P is used to fit the disturbance distribution Q (**Figure 2**). The algorithm aims to construct score indexes based on sJSD to detect a pre-disease state. First, the inconsistency index (ICI) based on JSD theory is constructed to calculate the difference in probability distributions between reference samples and case samples in different states. When complex diseases approach a critical state, the score of inconsistency index will convey an early warning signal (**Figure 2**), that is, the score will show a sudden upward trend at the critical transition to identify the pre-disease state. During this procedure, a group of molecules extracted from a genome-wide scale, which is more sensitive and active than other biomolecules for the arrival of the critical state’s early warning signal, known as sJSD signal biomarkers, can be used for further functional analysis and practical application.

This approach has been validated in five real disease datasets, including two genitourinary cancers, i.e., prostate cancer and bladder urothelial carcinoma (BLCA), influenza, Cervical squamous cell carcinoma (CESC) and pancreatic adenocarcinoma (PAAD), in which the prostate cancer dataset (GSE5345) and influenza dataset (GSE30550) are from the NCBI GEO database, BLCA, CESC and PAAD are from the cancer genome atlas (TCGA) database. The critical state or pre-disease state determined by sJSD is consistent with the observation in the real experiment, and the comparison of survival analysis before and after the critical state is significant. Furthermore, the sJSD signal biomarkers have been validated by functional enrichment.

## Materials and Methods

### Data Progression and Functional Analysis

The sJSD algorithm proposed in this paper has been applied to five datasets, including prostate cancer dataset (GSE5345), influenza virus infection time series data (GSE30550) from the GEO database (http://www.ncbi.nlm.nih.gov/geo) and bladder urothelial carcinoma (BLCA), cervical squamous cell carcinoma and endocervical adenocarcinoma (CESC) and pancreatic adenocarcinoma (PAAD) from the cancer genome atlas (TCGA) database (http://cancergenome.nih.gov).

The gene function annotation of each dataset is obtained through GeneCards (http://www.genecards.org/). The access to Enrichment is through the use of online analysis tools used in the Gene Ontology Consortium (GOC, http://geneontol-ogy.org), DAVID Bioinformatics Resources 6.8 (https://david.ncifcrf.gov/) and Circos (http://www.circos.ca/). PPI networks are drawn with the use of the online service web page STRING (https://string-db.org/) and the client software Cytoscape (https://cytoscape.org/).

### Background

DNB is a strongly correlated molecular subnetwork, whose molecules/variables dynamically change or fluctuate without keeping constant values in the pre-disease state (7). DNB has been applied in the analysis of real biological and clinical data in many research areas. However, it usually requires multiple samples, which limits its wide application due to unavailability of multiple samples on an individual for many cases. The single-sample Kullback-Leibler Divergence (sKLD) is proposed to quantify the single case sample disturbance on the background distribution (13) to solve the small sample problem, and the KL-divergence between two distributions can be written as

$$D_{KL}(P|Q)=\Sigma_{j}^{n}p(x_{j})log\frac{p(x_{j})}{\frac{1}{2}p(x_{j})+\frac{1}{2}q(x_{j})}.$$

However, KLD has the characteristics of non-negativity and asymmetry. In particular, if two distributions *P* and *Q* differ greatly and do not overlap at all, then KLD is meaningless and cannot be used.

This paper proposed the sJSD algorithm based on the Jensen-Shannon Divergence (JSD) theory (14). JSD is usually used to measure the difference of two probability distributions. It also lays a theoretical foundation for feature description (15) and difference measurement (16, 17). JSD is defined as follows

$$D_{JS}(P|Q)=\frac{1}{2}D_{KL}(P\left|\frac{P+Q}{2})+\frac{1}{2}D_{KL}(Q\right|\frac{P+Q}{2}).$$

Obviously *D_JS_(P*││*Q)=D_JS_(Q││P,)* and also *D_JS_(P*││*Q)=0* if and only if *P=Q.*

Among the applications of JSD, as mentioned in some references and literatures, JSD was used as a measure of the difference between two probability distributions. The probability distributions usually mean the probability density function or the probability distribution function (or the cumulative distribution function). Obviously, the probability density function represents the change rate of the probability for a random variable *x*, instead of the probability itself. While the probability distribution function represents the cumulative probability. In our paper, we use the probability distribution function, which expresses the integral area of the probability density function in the interval (*-∞,x_ij_*), where *x_ij_* is the expression of gene *g_i_* in the samples j. It not only makes full use of gene expression data, but also reflects the distribution characteristics of genes.

### Algorithm to Identify the Critical State Based on sJSD

The sJSD aims to detect critical state or pre-disease state of a complex disease and identify dynamic network biomarkers (DNBs) highly associated with disease deterioration based on single-sample of an individual. Given some reference samples (samples from a normal cohort that are viewed as background that represents the healthy or relatively healthy individuals), we can identify a critical state of the disease based on an individual’s single-sample data using the following algorithm (**Figure 2**).

[Step 1] Give reference samples and case samples.

The reference samples may be from healthy individuals, healthy tissues, or samples that have not developed any lesions at the beginning of the diseases. Each case sample refers to the single-sample of an individual during a disease process. In the datasets GSE30550 and GSE5345 taken from the GEO database, the reference samples refer to the samples at the initial time point of the experiment, while each case sample refers to the sample of an individual or subject during the experiment. In the datasets of BLCA, CESC, and PAAD from the TCGA database, reference samples refer to the samples of tumor-adjacent samples (relatively healthy samples), and case samples refer to the samples of tumor tissues at different stages of cancer development.

[Step 2] Fit Gaussian distributions of every gene based on reference samples and case samples of an individual.

Fit two Gaussian distributions of each gene in terms of the expression respectively from the reference samples and case samples, i.e., reference distribution and perturbed distribution.

For gene *g_i_*(*i*=1,...,*m*), its reference distribution *P_i_* (mean *µ_i_* and standard deviation *σ_i_*) is fitted based on the *n* expressions of *g*_i_ in the reference samples {*S_1_*,...,*S_j_*,...,*S_n_*} and the perturbed distribution *Q_i_* (mean ${\bar{\mu }}_{i}$and standard deviation ${\bar{\sigma }}_{i}$) is fitted based on expressions of *g*_i_ in the case samples $\left\{S_{T_{1}},\ldots ,S_{T_{t}},\ldots ,S_{T_{S}}\right\}$.

[Step 3] Transform gene expressions into probability distribution.

*s* samples *S*={*S_1,_S_2,...,_S_s_*} were randomly selected from *n* reference samples. The gene expression data in the reference samples and the gene expression data in the case samples are respectively transformed to cumulative probability *P_i_*(*x_ij_)* and $Q_{i}({\bar{x}}_{it})$.

$$P_{i}(x_{ij})=\frac{1}{\sigma_{i}\sqrt{2\pi }}{\int_{-\infty }^{x_{ij}}e}^{-\frac{{\left(u-\mu_{i}\right)}^{2}}{2\sigma_{i}^{2}}}du,$$

$$Q_{i}({\bar{x}}_{it})=\frac{1}{{\bar{\sigma }}_{i}\sqrt{2\pi }}{\int_{-\infty }^{{\bar{x}}_{it}}e}^{-\frac{{\left(u-{\bar{\mu }}_{i}\right)}^{2}}{2{\bar{\sigma }}_{i}^{2}}}du.$$

Where *x_ij_* is the gene expression data of gene *g_i_ (i=*1*,...,m)* in the reference samples *j* (*j*=1,...,*s*), *_it_* is the gene expression data of gene *g_i_* at time *t* (*t*=1,...,*s*) of the case samples for an individual.

[Step 4] Construct the inconsistency index based on JSD.

The dynamic differences between reference samples and case samples can be quantified when the reference distribution *P* is compared with the disturbance distribution *Q*, so as to reveal the critical state or pre-disease state of disease deterioration. The measurement $JS_{t}^{ICI}$calculated for every gene represents the difference of gene expressions between the normal samples and disease samples during disease processes.

$$JS_{t}^{ICI}(P_{i},Q_{i})=\frac{1}{2}D_{KL}(P_{i}|\frac{P_{i}+Q_{i}}{2})+\frac{1}{2}D_{KL}(Q_{i}|\frac{P_{i}+Q_{i}}{2}),$$

$$D_{KL}(P_{i},Q_{i})=\Sigma_{j=1}^{n}P_{i}(x_{ij})log\frac{P_{i}(x_{ij})}{\frac{1}{2}P_{i}(x_{ij})+\frac{1}{2}Q_{i}({\bar{x}}_{it})}.$$

We select the top 5% genes with the highest score at critical state as sJSD signal biomarkers, which are also the components of the DNBs. During the disease process, the sJSD signal biomarkers will move to the disease state first when the system experiences further disturbances of parameters into the disease stage. The comprehensive ICI score of global genes at time *t* is obtained,

$$D_{JS}^{ICI}(t)=\frac{\Sigma_{i}JS_{t}^{ICI}(P_{i},Q_{i})}{m},$$

which is in the range [0, ln2] for *log* base *e*. The higher the value $D_{JS}^{ICI}$of is, the greater the difference between the case sample and the reference sample is. Specifically, a abruptly increase of its score can be considered as an early-warning signal of critical transition during the disease progression (**Figure 2**).

## Results

In section 2, we showed the specific algorithm based on sJSD. With a sharp increase in ICI score being treated as a signal of the approaching critical state, we can detect complex disease’s critical state through a single case sample. A single sample with high-throughput data is regarded as the detection target used to identify early warning signals of complex disease’s critical transition. It is of great significance to identify the critical transition through the single sample of an individual since it is difficult to obtain extensive samples from an individual without any symptoms of diseases in the early stage of disease development. To describe how sJSD works, we applied the sJSD method to five real datasets, including influenza dataset (GSE30550), prostate cancer dataset (GSE5345) from GEO database (https://www.ncbi.nlm.nih.gov/geo/) and BLCA PAAD, CESC from the TCGA database (http://cancergenome.nih.gov). The sJSD method’s effectiveness in quantifying the tipping point before the critical transition into severe disease state was successfully verified by identifying pre-disease states of these datasets.

### Recognition of Critical State of Prostate Cancer

We apply the sJSD method to the microarray data of the dataset GSE5345. In this experiment, the synthetic androgen R1881 (experimental group) and alcohol (control group) were used to stimulate human prostate cell line LNCap to explore the effect of synthetic androgen R1881 on the gene expression level. In the original experiment, the case samples were derived from human prostate cancer cell lines stimulated by the synthetic androgen R1881 for 48 hours. The control samples were obtained from human prostate cancer cell lines stimulated by synthetic androgen R1881 for 0 hours. In the experiment, there are seven sampling time points (0, 6, 9, 12, 18, 24, 48h), and there are four samples at each time point (except six samples at the 6th hour) (18). The four samples at the first time point 0h are taken as reference samples.

**Figure 3:**
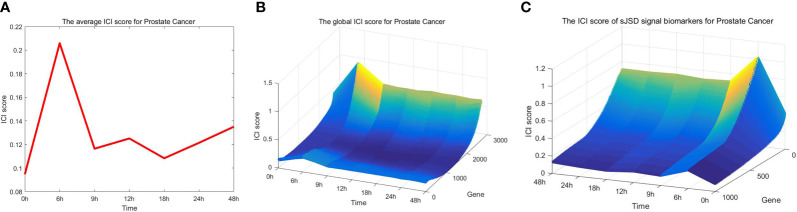
Application of sJSD method in prostate cancer. **(A)** The curve of average ICI score for prostate cancer. The average ICI score peaks at the 6th hour. It can be viewed as an early-warning signal of critical transition for prostate cancer. **(B)** The landscape of the global ICI score. The dynamical change of global ICI scores proves the arrival of the critical transition from a single gene’s perspective. **(C)** The landscape of ICI scores of sJSD signal biomarkers. The ICI scores of sJSD signal biomarkers increase significantly at the 6^th^ hour with a more intuitive landscape.

**Figure 4:**
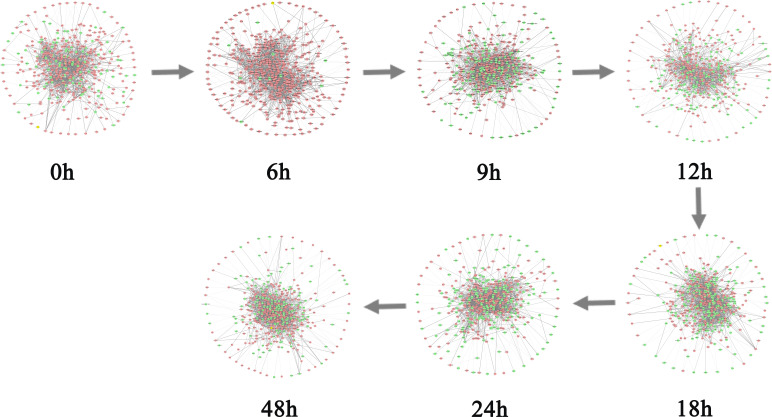
The dynamic evolution of sJSD signal biomarkers. The PPI network shows the dynamic structural changes of sJSD signal biomarkers, in which red circles represent sJSD signal biomarker genes and green circles represent non-sJSD signal biomarker genes. It can be seen that at the 6^th^ hour, the sJSD signal biomarkers become more active, and the network structure undergoes significant changes.

As can be seen in **Figure 3A**, the red curve is characterized by a sudden rise and reaches the peak at the 6th hour, indicating that the warning signal of pre-disease state is detected around the 6th hour of prostate cancer. After the 6th hour, the disease begins to deteriorate. For example, cancer may lead to bone metastasis at first, followed by lymphatic metastasis and even visceral metastasis with the deepening of lesions (19). **Figure 3B** and **Figure 3C** respectively show the landscapes of global ICI score and sJSD signal biomarkers, there are sudden rises of ICI scores at the 6th hour. We can see that the sJSD signal biomarkers can obtain the same results as an individual’s whole gene sequence when detecting the critical state. Furthermore, the landscape of sJSD signal biomarkers is more intuitive and sensitive for the signals of critical transitions. The result can provide a reference for medical diagnosis and effectively reduce gene sequencing cost in clinical practice. **Figure 4** shows the dynamic evolution of the Protein-Protein Interaction (PPI) network of sJSD signal biomarkers. In the 6th hour, the network structure changed significantly, which confirmed the critical transition from the molecular network level.

Next, we performed a functional analysis of sJSD signal biomarkers. By counting the six samples’ sJSD signal biomarkers at the critical state, we obtained the high-frequency JSD genes (**Supplementary Table S1**). The relationship between the high-frequency genes and the pathogenesis of prostate cancer or cancer are exhibited in **Table 1**. For instance, in the current sample, the genes GNAS, GNAQ, and GNA11 were widely altered across cancer types, and these alterations often were accompanied by specific genomic abnormalities in AURKA, CBL, and LYN (20). HDAC4 is recruited to the nuclei of HR cancer cells, where it may exert an inhibitory effect on differentiation and contribute to the development of the aggressive phenotype of late stage CaP (21). Filamin-B (FLNB) was identified as biomarkers in a strategy for prostate cancer (PrCa) biomarker discovery (22). Reduced expression of HSP90B1 was associated with apoptosis induction by androgen receptor and prostate specific antigen (23). Smad2 was found play a critical role in the basal epithelial or stem cell compartment of the prostate as a tumor suppressor (24). High NR2C2 expression was associated with nonfunctioning pituitary adenoma invasion, recurrence, and progression (25). The GSPT1/GSK pathway exerts tumor-promoting actions in colon cancer oncogenesis and progression. The GSPT1/GSK pathway may thus be an effective target for controlling colon cancer (26). Recently, LPP has emerged as a critical inducer of tumor cell migration, invasion and metastasis (27). The NRIF3/DIF-1/FASTKD2 pathway acts as a “death switch” in breast and prostate cancer cells, how FASTKD2 initiates the apoptotic response will allow for the development of therapeutic agents for the treatment of androgen-independent prostate cancer (28).

**Table 1:** The high frequency genes in 6 “sJSD signal biomarkers” groups at the critical stage (6th hour) for prostate cancer.

Gene Id	Gene name	Frequency	Location	Family	Relation with cancer progression
GNAQ	G Protein Subunit Alpha Q	6	plasma membrane	the G-alpha family	In the current sample, the gene GNAQ was widely altered across cancer types (20).
HDAC4	Histone Deacetylase 4	6	nucleus	HD type 2 subfamily	HDAC4 is recruited to the nuclei of HR cancer cells, where it may exert an inhibitory effect on differentiation and contribute to the development of the aggressive phenotype of late stage CaP (21).
FLNB	Filamin B	6	plasma membrane	the filamin family	Filamin-B (FLNB) was identified as biomarkers in a strategy for prostate cancer (PrCa) biomarker discovery (22).
HSP90B1	Heat Shock Protein 90 Beta Family Member 1	6	endoplasmic reticulum	the heat shock protein 90 family	Reduced expression of HSP90B1 was associated with apoptosis induction by androgen receptor and prostate specific antigen (23).
SMAD2	SMAD Family Member 2	6	nucleus	The SMAD family	Smad2 was found play a critical role in the basal epithelial or stem cell compartment of the prostate as a tumor suppressor (24).
NR2C2	Nuclear Receptor Subfamily 2 Group C Member 2	6	nucleus	NR2 subfamily	High NR2C2 expression was associated with nonfunctioning pituitary adenoma invasion, recurrence, and progression (25).
GSPT1	G1 To S Phase Transition 1	6	cytosol	Classic translation factor GTPase family	The GSPT1/GSK pathway exerts tumor-promoting actions in colon cancer oncogenesis and progression (26).
LPP	LIM Domain Containing Preferred Translocation Partner In Lipoma	6	plasma membrane	the zyxin/ajuba family	LPP has emerged as a critical inducer of tumor cell migration, invasion and metastasis (27).
FASTKD2	FAST Kinase Domains 2		mitochondrion	the FAST kinase family	The NRIF3/DIF-1/FASTKD2 pathway acts as a “death switch” in breast and prostate cancer cells, how FASTKD2 initiates the apoptotic response will allow for the development of therapeutic agents for the treatment of androgen-independent prostate cancer (28).

**Figure 5:**
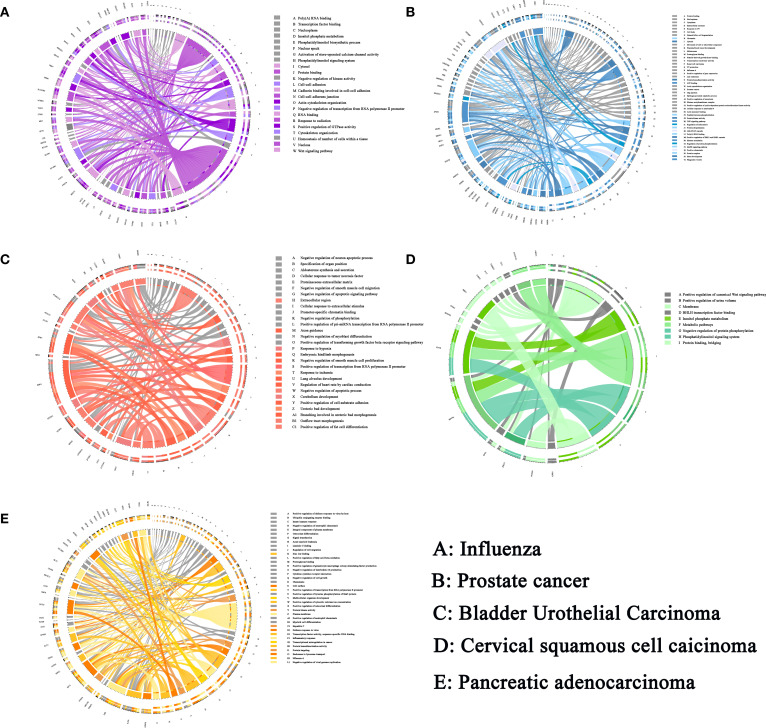
The sJSD signal biomarkers are involved in important biological processes in five datasets. **(A)** The GO and KEGG enrichment pathway of influenza. **(B)** The GO and KEGG enrichment pathway of prostate cancer. **(C)** The GO and KEGG enrichment pathway of BLCA. **(D)** The GO and KEGG enrichment pathway of CESC. **(E)** The GO and KEGG enrichment pathway of PAAD. The left side of the outer ring represents sJSD signal biomarkers detected by our algorithm and the right side of the outer ring represent detailed biological processes in which these genes are involved. In the inner ring, the Color and width of links respectively indicate diverse enrichment pathway and significant levels of genes function.

The GO analysis’ functional enrichment shows that the high-frequency JSD signaling genes are involved in biological processes (**Table 2**), including nuclear-transcribed mRNA catabolic process (GO:0000184), positive regulation of transcription (GO:0045893), DNA biosynthetic process (GO:0071897) and cell-cell adhesion (GO:0098609), etc. These biological processes are closely related to the progression of prostate cancer. The enrichment analysis of KEGG signaling pathways shows that the high-frequency genes are mainly involved in signaling pathways (**Table 2**) such as the GTPase activator activity, GnRH signaling pathway, pathways in cancer and inflammatory mediator regulation of TRP channels, etc. **Figure 5B** shows the significance level of each high-frequency gene in the sJSD signal biomarker groups which is enriched to the biological process.

**Table 2:** The functional enrichment of high-frequency “sJSD signal biomarkers” at the critical stage samples for prostate cancer.

Gene Ontology Consortium			KEGG	
**enriched biological process**	**enriched p value**		**enriched biological process**	**enriched p value**
nuclear-transcribed mRNA catabolic process (GO:0000184)	0.010119341		GTPase activator activity	0.089337462
positive regulation of transcription (GO:0045893)	0.043717073		GnRH signaling pathway	0.009172748
DNA biosynthetic process (GO:0071897)	0.09317234		Pathways in cancer	0.037572134
cell-cell adhesion (GO:0098609)	0.018785728		Salmonella infection	0.059682054
cellular response to mechanical stimulus (GO:0071260)	0.00239874		Inflammatory mediator regulation of TRP channels	0.079769321
mitotic chromosome movement towards spindle pole (GO:0007079)	0.014924228		Estrogen signaling pathway	0.081176119
positive regulation of protein sumoylation (GO:0033235)	0.040515966		Chagas disease (American trypanosomiasis)	0.088325936

### The Critical State of Individual Influenza Infection

We applied the sJSD method to individual time-series datasets GSE30550 (29), which contains the samples of 17 volunteers. They were infected with H3N2/Wisconsin virus in their nasal cavity, nine of them (subject 1, 5, 6, 7, 8, 10, 12, 13, and 15) subsequently developed severe infection symptoms, while the other eight subjects remained healthy. In the subsequent analyzes, samples from volunteers with severe flu-like symptoms were treated as case samples, and those who remained healthy were treated as reference samples. The volunteers’ peripheral blood was collected about every eight hours, for 108 hours from the infection time, to measure gene expression profiles. The samples of 8th volunteer at the 21th hour, the 13th volunteer at the 24th and 36th hour, and the 17th volunteer at the 36th hour have been lost. The other volunteers have 16 sampling time points (-24, 0, 5, 12, 21, 29, 36, 45, 53, 60, 69, 77, 84, 93, 101, 108 h), a total of 268 pairs of gene expression data. Each individual has only one sample data at each time point. For each volunteer, the gene expression profile at the previous two points (-24, 0h) is viewed as reference samples.

**Figure 6:**
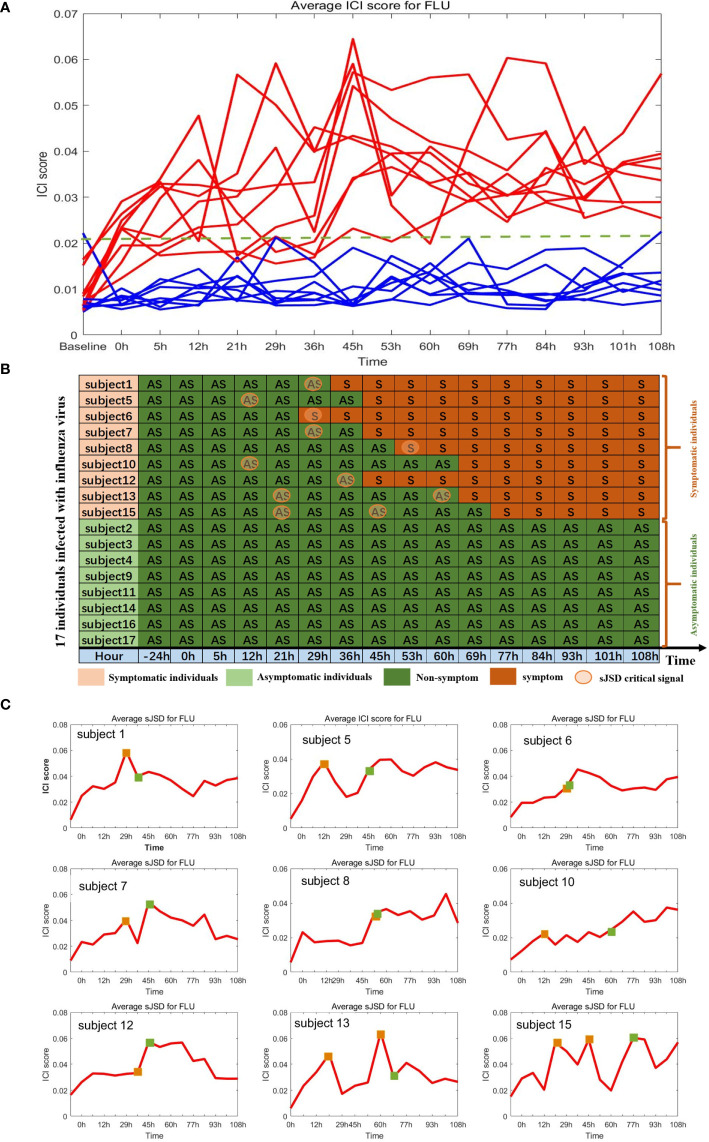
Identification of critical state of influenza based on sJSD. **(A)** The ICI score curve for 17 subjects. The red curve represents the average ICI scores for 9 symptomatic subjects. The blue curve represents the average ICI score for eight asymptomatic subjects. **(B)** Summarized predicted results. For each symptomatic individual, the ICI scores successfully indicate the impending flu symptoms. While for asymptomatic individuals, there were no wrong warning signals. **(C)** The curve of average ICI scores for nine symptomatic individuals. The green squares represent the initial time when flu symptoms appear (clinically observed), and the orange squares represent the critical state identified by the ICI score.

By applying the algorithm proposed in section 2, we obtained the ICI score of each gene for each sample at different time points, in which the ICI scores of symptomatic subjects showed significant changes, while those of asymptomatic subjects did not (**Figures 6A, B**). The ICI scores of nine subjects with flu symptoms are shown in **Figure 6C**. The dramatic increase of ICI Score successfully provided an early warning signal of the upcoming disease state. Specifically, in subjects 13 and 15, two warning signals have been detected before the onset of flu symptoms. All the influenza warning signals in nine symptomatic individuals were successfully detected for the upcoming onset of symptoms, and no wrong warning signals were detected in eight asymptomatic individuals. Hence, the sJSD algorithm can effectively identify the critical state and accurately detect the early warning signal for every individual with influenza virus infection. The average ICI Score of all genes is defined as the global ICI Score. A sudden increase of global ICI score at the genome-wide scale indicates an early warning signal of the individual’s critical transition. To study the critical biomarkers of complex diseases, we mapped the landscape based on the average ICI score of the top 5% genes with the highest ICI score. The results showed that these biomarker genes’ landscapes were more intuitive and significant than that of the global genes in detecting complex diseases’ critical state. Therefore, we selected the top 5% genes of ICI score at the critical stage as JSD signaling biomarkers (**Supplementary Table S0**).

The sJSD signal biomarkers vary among individuals even in the case of the same disease, the high-frequency genes in 9 sJSD signal biomarker groups are shown in **Supplementary Table S0**. We carried out the functional enrichment of 365 high-frequency genes in the 9 JSD signal biomarker groups. Through Gene Ontology (GO) analysis, 153 GO sets are significantly up-regulated, linked with the influenza virus’ infection process (**Figure 5A**). Among them, 23 cell components are significantly up-regulated, including nucleoplasm (GO:0005654), cytosol (GO:0005829) and nucleus (GO:0005634), etc. 82 biological processes are significantly up-regulated, including positive regulation of transcription (GO:0045893), viral process (GO:0016032), phosphatidylinositol-mediated signaling (GO:0048015), and Wnt signaling pathway (GO:0016055), etc. There are 48 significantly up-regulated molecular functions, including protein binding (GO:0005515) and transcription coactivator activity (GO:0003713), etc. The Kyoto Encyclopedia of Genes and Genomes (KEGG) signaling pathways mainly include epstein-Barr virus infection, herpes simplex infection, bacterial invasion of epithelial cells and pathogenic Escherichia coli infection, etc. These significant gene sets and pathways can be used as candidate pathways related to influenza virus infection, and researchers can detect SNP typing on these gene sets to guide the early diagnosis, prevention, and personalized treatment of influenza.

### Critical Transition of the Tumor Datasets

To demonstrate the applicability of the sJSD approach, we applied it to three tumor datasets to detect critical states of different diseases, including bladder urothelial carcinoma (BLCA), cervical squamous cell carcinoma and endocervical adenocarcinoma (CESC), and pancreatic adenocarcinoma (PAAD) from the cancer genome atlas (TCGA). Each disease dataset includes tumor and tumor-adjacent samples. The tumor samples can be divided into different states according to the samples’ clinical information. The tumor samples of BLCA are divided into four states, tumor samples of CESC are divided into six states, and tumor samples of PAAD are divided into five states. In the three datasets, tumor-adjacent samples are considered reference samples or reference data. The ICI was applied to calculate for each tumor sample based on the proposed algorithm sJSD, every stage’s average ICI score was used to characterize the possible critical state.

Through the sJSD algorithm, we successfully detected the critical state and obtained the sJSD signal biomarkers (**Supplementary Table S2**). Besides, we got the ICI scores of genes in the tumor samples at different stages. Firstly, the ICI scores of genes at different tumor stages are calculated to screen sJSD signal biomarkers, obtaining the high-frequency genes (**Supplementary Table S1**) that appeared in multiple sJSD signal biomarker groups. Secondly, the average ICI scores of sJSD signal biomarkers at different stages can be used to detect the early warning signals of the critical transition of complex diseases. Finally, a PPI network was drawn to illustrate the dynamic structural changes of sJSD signal biomarkers from the molecular network level.

**Figure 7:**
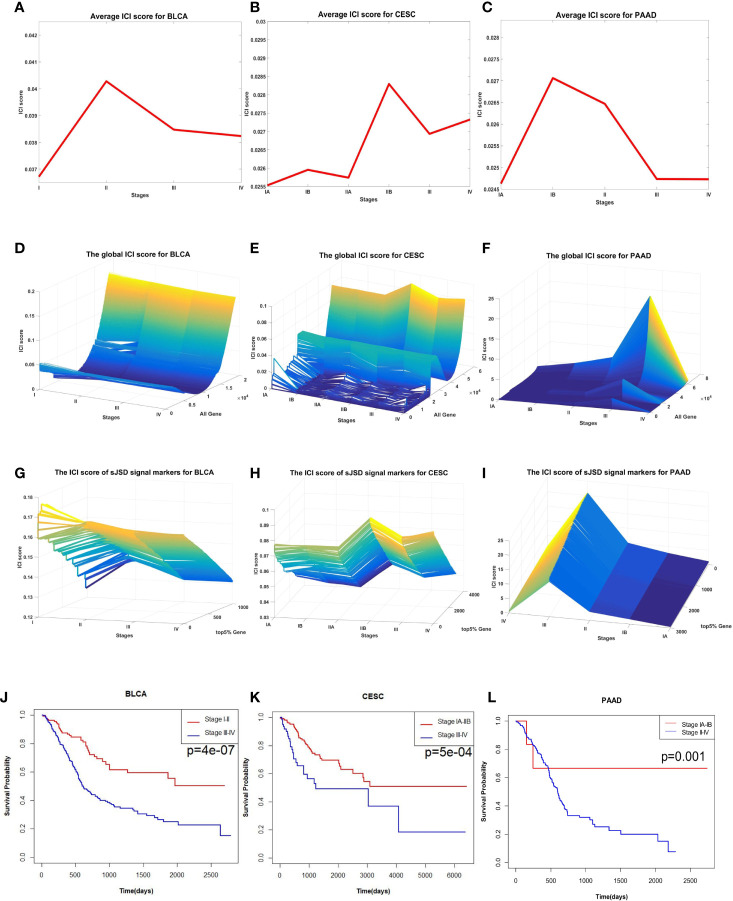
The application of sJSD in three cancers. Identification of critical transitions in cancer progression: **(A)** BLCA, **(B)** CESC, **(C)** PAAD. The landscapes of global ICI scores for three cancers: **(D)** BLCA, **(E)** CESC, **(F)** PAAD. The landscapes of ICI scores of sJSD signal biomarkers for three cancers: **(G)** BLCA, **(H)** CESC, **(I)** PAAD. Comparison of survival curves for three cancers before and after the critical state: **(J)** BLCA, **(K)** CESC, **(L)** PAAD.

As shown in **Figures 7A–I**, significant changes in ICI scores indicated the critical state of three cancers prior to tumor metastasis. The dynamic changes of average ICI score for all the three cancers are shown in **Figures 7A–C**, the landscapes of global ICI score for three cancers are presented in **Figures 7D–F**, and the landscapes of ICI score of sJSD signal biomarkers are exhibited in **Figures 7G–I**. To validate the identified critical state. The Kaplan-Meier (log-rank) prognosis analysis was performed based on the samples before and after the critical state’s onset (**Figures 7J–L**). Normally, after diagnosis, there is a higher survival expectation before identifying the critical state, but there is a lower survival expectation after identifying the critical state. However, there was no significant difference in prognostic analysis before and after the remaining states (**Supplementary Materials A**).

#### The Critical State of BLCA

Bladder cancer is one of the common diseases of the urinary system (30), and bladder urothelial carcinoma is the most common type of bladder cancer. Its main treatment is surgical resection. However, the disease is easy to recur after surgery, so the prognosis is generally poor. Specifically, the lack of effective biomarkers makes it difficult to illuminate the pathogenesis of BLCA (31). Therefore, detecting the early warning signals of the onset for BLCA and identifying critical biomarker genes have great significance for disease prevention.

**Figure 8:**
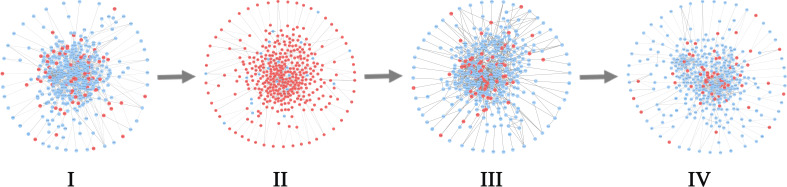
The dynamic evolution of sJSD signal biomarkers for BLCA. There is a significant change in the internal structure of the sJSD signal biomarkers at stage II, in which red circles represent sJSD signal biomarker genes and blue circles represent non-sJSD signal biomarker genes.

For BLCA, 405 tumor samples and 19 tumor-adjacent samples were obtained from TCGA. Based on the clinical information of each sample, cancer samples were divided into four stages, namely Stage I (2 samples), Stage II (130 samples), Stage III (139 samples), and Stage IV (134 samples) of BLCA. As shown in **Figure 7A**, the sudden rise of ICI scores indicates the impending critical transition after the critical state (Stage II). Upper urinary tract hydrops or related symptoms caused by tumor compression and invasion are common to appear at stage III, after which distant metastasis of cancer occurs and most patients have a pelvic recurrence (32). The landscape in **Figure 7D** shows the dynamic change of the global ICI score for BLCA. It can be seen that there is a significant increase at stage II. Besides, the landscape of ICI scores of sJSD signal biomarkers is shown in **Figure 7G**, which is particularly sensitive to the arrival of critical transition signals. **Figure 8** shows the dynamic evolution of the sJSD signal biomarkers’ network structure, in which there are 500 nodes and 1,199 edges. This group of molecules is extremely active and highly correlated at Stage II and can be regarded as signal biomarkers to detect the critical state before disease deterioration. This critical state has been validated by survival analysis. **Figure 7J** shows that the survival time of samples from Stages I-II is longer than that of samples from Stages III-IV, represented with a significant value P = 0.001 for both types of samples. It indicates that the detection of the critical state is significant for patients’ survival and can be applied in clinical practice to provide a reference for disease diagnosis. To verify other critical states’ existence affecting survival time, a series of prognostic analyzes were performed for other stages, such as Stage I and stage II-IV, stage I-III and Stage IV (**Supplementary Materials A1**). The results showed no significant change in the survival time of the samples before or after other states. In other words, there is no other critical stage prior to the critical transition (Stages I) or after the critical transition (Stages III-IV). The sJSD method successfully detected critical transitions related to disease progression and survival time in BLCA.

The high-frequency genes in the sJSD signal biomarker groups of BLCA have been found to be related to the pathogenesis of BLCA in some literatures (**Table 3**). The decreased expression of RELN was associated with increased migratory ability, reduced survival, and poor prognosis (33). LRRC2 was found to be localized to the mitochondria in human cells and transcriptionally regulated by the mitochondrial master regulator Pgc-1α (34). HAND2-AS1 declined in bladder cancer and correlated negatively with invasion and grades (35). FXYD6 may be a new biomarker for cancer and may be associated with a favorable prognosis in this malignant disease (36). Ectopic expression of RBMS3 markedly suppressed cell proliferation and clonogenicity and promoted apoptosis *in vitro* (37). WISP2 overexpression inhibited cell growth and induced cell apoptosis, suppressed cell migration, and invasion in cells (38). HLF is a novel oncofetal protein that is reactivated in HCC by SOX2 and OCT4 (39). The results of GO analysis showed that the high-frequency genes at the critical state are involved in the following biological processes (**Supplementary Materials B1**), such as adenylate cyclase-modulating G-protein coupled receptor signaling pathway (GO:0007188), the apoptotic process involved in heart morphogenesis (GO:0003278), and collagen fibril organization (GO:0030199). The enrichment analysis of KEGG signal pathways (**Supplementary Materials B1**) shows that the high-frequency genes are involved in Adrenergic signaling in cardiomyocytes, Estrogen signaling pathway and cAMP signaling pathway, etc. These biological processes and signaling pathways are closely related to the deterioration of BLCA. The significance level of each high-frequency genes that enriched to the biological process is shown in **Figure 5C**.

**Table 3:** The high frequency genes in 130 “sJSD signal biomarkers” groups at the critical stage (stage II) for BLCA.

Gene Id	Gene name	Frequency		Location	Family	Relation with cancer progression
RELN	Reelin	65	Extracellular		other	The decreased expression of RELN was associated with increased migratory ability, reduced survival and poor prognosis (33).
LRRC2	Leucine Rich Repeat Containing 2	53	Nucleus		transcription Factor	LRRC2 was found to be localized to the mitochondria in human cells and transcriptionally-regulated by the mitochondrial master regulator Pgc-1α (34).
HAND2	Heart And Neural Crest Derivatives Expressed 2	44	Nucleus		transcription Factor	HAND2-AS1 declined in bladder cancer and correlated with the depth of invasion and grades negatively (35).
FXYD6	FXYD Domain Containing Ion Transport Regulator 6	40	Plasma membrane		Protein Coding gene	FXYD6 may be a new biomarker for cancer and may be associated with a favorable prognosis in this malignant disease (36).
RBMS3	RNA Binding Motif Single Stranded Interacting Protein 3	37	Nucleus		Protein Coding gene	ectopic expression of RBMS3 markedly suppressed cell proliferation and clonogenicity and promoted apoptosis *in vitro (* 37).
WISP2	WNT1 inducible signaling pathway protein 2	35	Extracellular space		other	WISP2 overexpression inhibited cell growth and induced cell apoptosis, suppressed cell migration and invasion in cells (38).
HLF	HLF Transcription Factor	34	Nucleus		transcription Factor	HLF is a novel oncofetal protein which is reactivated in HCC by SOX2 and OCT4 (39).

#### The Critical State of CESC

CESC is the second most common malignant tumor, thus being a serious threat to women’s health and life (40). The main pathological types of cervical cancer include cervical squamous cell carcinoma and cervical adenocarcinoma. Due to the lack of reliable diagnostic and prognostic biomarkers, the prognosis of patients with CESC is unsatisfactory (41). Therefore, early diagnosis and identification of critical biomarkers are crucial to improving patients’ survival rate with CESC.

**Figure 9:**
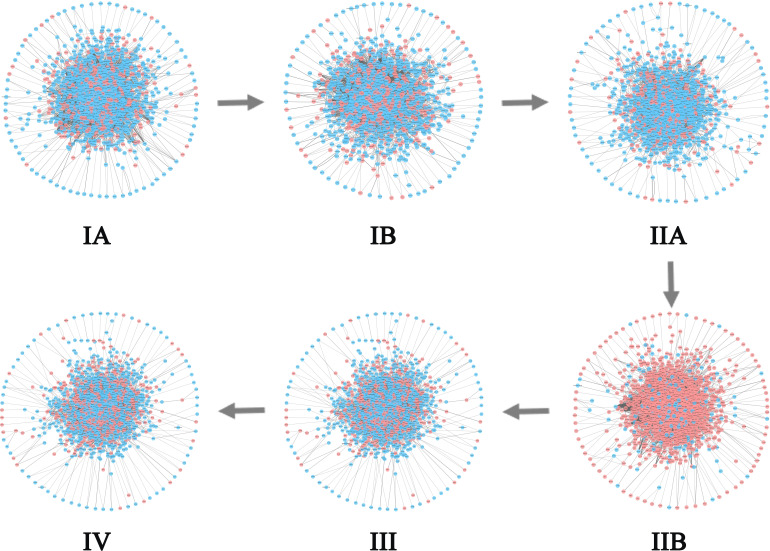
The dynamic evolution of sJSD signal biomarkers for CESC. It can be seen that there is a significant change in the internal structure of sJSD signal biomarkers at stage IIB.

For CESC, 299 tumor samples and three tumor-adjacent samples were obtained from TCGA. According to the samples’ clinical information, tumor-adjacent samples were divided into six stages (IA, IB, IIA, IIB, III, IV). As shown in **Figure 7B**, ICI scores suddenly rise at Stage IIB, indicating a critical transition at Stage IIB. After this transition, the tumor begins to infiltrate deep into the cervical tissue, invading the surrounding blood vessels and lymphatic vessels, and worsening the prognosis (42). **Figure 7E** shows the landscapes of the global ICI score of genes, **Figure 7H** shows the landscape of ICI scores of sJSD signal biomarkers. Similarly, both of the ICI scores peaked at stage IIB, which demonstrates the emergence of **c**ritical transition from the molecular level. Compared with the landscape of global ICI score, the landscape of ICI scores of sJSD signal biomarkers is more intuitive, which indicates that sJSD signal biomarkers are highly sensitive to the warning signal of the tipping point. **Figure 9** exhibits the PPI network of sJSD signal biomarkers, in which the network structure presents significant changes at Stage IIB. To further verify the critical state, the prognostic analysis was performed on the samples before and after Stage IIB (**Figure 7K**). The samples at Stage IA-IIB have a longer survival time and a higher survival probability than the samples at Stage III-IV (significant value P = 0.001). If the early warning signal of critical transition can be diagnosed at the critical state opportunely, people will have a better prognosis. The comparison of prognostic analysis before and after other stages are presented in **Supplementary Materials A2**.

Through the sJSD algorithm, we successfully detected the early warning signals of critical transition for CESC and identified the sJSD signal biomarkers (**Supplementary Table S2**) indicating critical state’s arrival. The high-frequency genes in sJSD signal biomarker groups of CESC have been related to CESC’s pathogenesis in some literatures (**Supplementary Materials B2**). Zim2 is found to be a new mutant gene signaling cancer (42). The cytodomain of PCDH11X has been shown to interact with β‐catenin, inducing the Wnt signaling pathway in cultured cancer cells (43). AGBL4 is identified as a specific gene for cancer (44). DACH2 is an independent prognostic biomarker that can be used at the initial diagnosis of cancer (UCB) to identify patients with a high potential to develop metastasis (45). EphA5 is abnormally expressed in numerous malignant tumors and may be involved in cancer’s radiosensitivity (46). MiRNAs could inhibit LINC01016 transcription, forming two reciprocal repression cycles, which influence cancer cells’ biological behavior (47). PGM5-AS1 is downregulated in human colorectal cancer tissues and cells (48). Besides, The GO analysis shows that high-frequency genes (**Supplementary Table S1**) of CESC are involved in the following biological processes (**Figure 5D**), axon Guidance (GO:0007411), enzyme Binding (GO:0019899), focal adhesion (GO:0005925), etc. The enrichment analysis of KEGG signaling pathways (**Figure 5D**) shows the high-frequency genes are primarily involved in Dilated cardiomyopathy (DCM), Hypertrophic cardiomyopathy (HCM), and cGmp-Pkg signaling pathway, etc. These biological processes and signaling pathways are closely related to the deterioration of CESC.

#### The Critical State of PAAD

PAAD is a highly invasive malignant tumor of the digestive system, and its occurrence and mortality continue to increase (49). The early clinical manifestations of PAAD are not obvious, while the tumor develops rapidly and the degree of malignancy is extremely high. The cancer is already in the locally advanced stage or occurs distant metastasis when people show specific symptoms (50). Therefore, it is of great significance to explore the critical transition and critical biomarkers before the deterioration of PAAD for early diagnosis and survival rate improvement.

**Figure 10:**
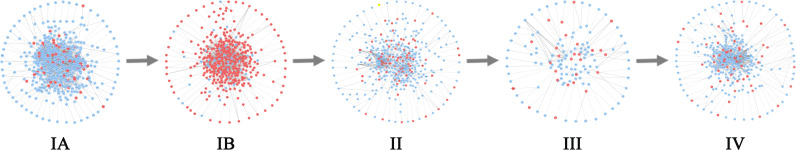
The dynamic evolution of the sJSD signal biomarkers for PAAD. It can be seen there is a significant change in the internal structure of sJSD signal biomarkers at stage IB.

For PAAD, there were 175 tumor samples and four tumor-adjacent samples from TCGA. According to the samples’ clinical information, the tumor-adjacent samples were divided into six stages, i.e., Stage IA, Stage IB, Stage IIA, Stage IIB, Stage III, and Stage IV. In **Figure 7C**, the average ICI scores peak at Stage IB suggesting that there is a critical transition after Stage IB for PAAD. Patients in Stage IIA-III are prone to having symptoms, such as abdominal or lower back pain, tumor with surrounding tissue invasion, even the nerve tissue may be infiltrated by the pancreatic tumor (Stage IV) (51). **Figure 7F** shows the landscape of global ICI scores of PAAD, the ICI scores of some genes increase significantly at Stage IB, which indicates the emergence of the critical transitions of PAAD. It can be seen from **Figure 7I** that there is a more significant rise in the landscape of ICI scores of sJSD signal biomarkers at Stage IB. Therefore, the sJSD algorithm can detect the critical state of complex diseases and identify the sJSD signal biomarkers that indicate the critical state. The dynamic evolution of network structure in sJSD signal biomarkers is shown in **Figure 10**, the network structure changed significantly at Stage IB. See the **Supplementary Table S2** for details of sJSD signal biomarkers. As shown in **Figure 7L**, there is a significant difference between the survival times of Stage IA-IB (red curve) and the stage II-IV (blue curve). Before the tipping point, patients’ survival time is significantly longer than the patients after the tipping point. In addition, there are no significant differences in the survival time of samples before and after other stages (**Supplementary Materials A3**). The result indicates that the sJSD successfully detected the early warning signals of critical transitions of survival time and distant metastasis at Stage IB.

The high-frequency genes in the sJSD signal biomarker groups of PAAD are closely related to pathogenesis **Supplementary Materials B3**). For instance, compared to the second sample, HNRNPCL1 in the first sample indicates an increased probability of suffering from pancreatic cancer (52). LINC00682 methylation is associated with recurrence and decreased overall survival in HCC patients (53). LINC01180 has a role in physiological and pathological processes, including cancer (54). MORC is expressed in 36% of ten CT genes (The Cancer-testis (CT) antigens are expressed in many malignant tumors) (55). MiR-656 influences the proliferation and migration of cancer-related cells (56). LINC00906 is involved in cellular differentiation and proliferation as post-transcriptional regulators of splicing or as molecular decoys for miRNA (57). RGPD6, a transcription factor, is the most mutated gene in tumors (58). MiR-1250 is located in 17 q25.3, whose genetic phenotype is often closely related to malignant biological behavior such as vascular invasion and distant metastasis of tumors (59). Besides, the GO analysis shows that high-frequency genes (**Supplementary Table S1**) of CESC are involved in the following biological processes (**Figure 5E**), such as immune system process (GO:0002376), regulation of immune system process (GO:0002682), cell surface receptor signaling pathway (GO:0007166). KEGG path analysis shows that sJSD signal biomarkers are found to be involved in some signal pathways (**Figure 5E**), including Allograft Rejection signaling pathway, Cytokine receptor interaction signaling pathway, graft-verse-host disease signaling pathway, and others. These biological processes and signaling pathways are closely related to the deterioration of PAAD.

### Revealing Non-Differential ‘Dark Genes’ by sJSD Method

In clinical practice and scientific research, differentially expressed genes draw much attention in early diagnosis of disease, screening drug targets, treating diseases, and developing new drugs. However, some non-differential genes in the coding region of DNA are called “Dark Matter” (60). Based on sJSD methods, we found some “dark genes” without differential expressions which are especially sensitive to ICI score. Traditional analyses usually ignore it. The ‘dark genes’ and differentially expressed genes of three tumor datasets are respectively shown in **Supplementary Table S4** and **Supplementary Table S3**.

To further explore “dark genes”, we focused on their role in cancer prognosis of BLCA, CESC, and PAAD. Firstly, we selected sJSD signal biomarkers (top 5%) genes with the highest ICI score that are not differentially expressed. Secondly, we analyzed the prognosis of these “dark genes” respectively based on gene expression and ICI score by dividing the samples into two groups based on the median of genes expression or ICI score, in which Group 1 is a group with higher value and Group 2 is a group with a lower value. Thirdly, based on the result of prognosis, the “dark genes” could be categorized into two types of molecules as a mutual biomarker for all samples. Those genes with high scores that cause poor prognosis were termed “negative dark genes”, and those genes with high scores that cause good prognosis termed “positive dark genes”. If “negative dark genes” appeared in the sJSD signal biomarkers of a sample, the sample’s prognosis would be more negative than that of other samples. Similarly, if “positive dark genes” appeared in the sJSD signal biomarkers of a sample, the sample’s prognosis would be more positive than others.

For BLCA, further analysis showed that “dark genes” were all strongly related to patients’ survival based on ICI score but not expression levels. **Supplementary Material C1** shows the survival analysis of CASD1, FAM86B1, KRBA2, and the other four genes results with P-values < 0.05 based on some non-differential genes in sJSD signal biomarker groups of BLCA. A higher level of ICI score in CASD1, FAM86B1, KRBA2, and 43892 is significantly related to a good prognosis, i.e., positive “dark genes”. While a higher level of ICI score in C15orf52, KRBA1, and UBBE2D4 is significantly related to poor prognosis, i.e., negative “dark genes” (**Table 4**). This confirmed the effectiveness of the development of BLCA for the ‘dark gene’ in the sJSD signaling biomarker groups.

**Table 4:** ‘Dark genes’ representing positive and negative biomarkers for BLCA, CESC and PAAD disease states.

BLCA		CESC		PAAD	
**Positive dark genes**	**Negative dark genes**	**Positive dark genes**	**Negative dark genes**	**Positive dark genes**	**Negative dark genes**
CASD1	C15orf52	ZNF487	MAGEL2	VIM-AS1	
FAM86B1	KRBA1	EEF1A1P9	ANKHD1-EIF4EBP3	FLJ38122	
KRBA2	UBBE2D4	C1QTNF9		PAICSP1	
43892		FAM66D		HLA-DQB1-AS1	
		MFRP		RNU6ATAC16P	
				RNU6-658P	
				LINC00619	
				AL590762.11	

For CESC, **Supplementary Material C2** shows the survival analysis of MAGEL2, ZNF487, EEF1A1P9, and the other four genes results with P-values < 0.05 based on some non-differential genes in sJSD signal biomarker groups of CESC. A higher level of ICI score in “positive dark genes”, i.e., ZNF487, EEF1A1P9, C1QTNF9, FAM66D, and MFRP, is significantly related to a good prognosis. While a higher level of ICI score in ‘negative dark genes’, i.e., MAGEL2 and ANKHD1-EIF4EBP3, is significantly related to poor prognosis (**Table 4**). This validated the effectiveness of the development of CESC for the ‘dark gene’.

**Figure 11:**
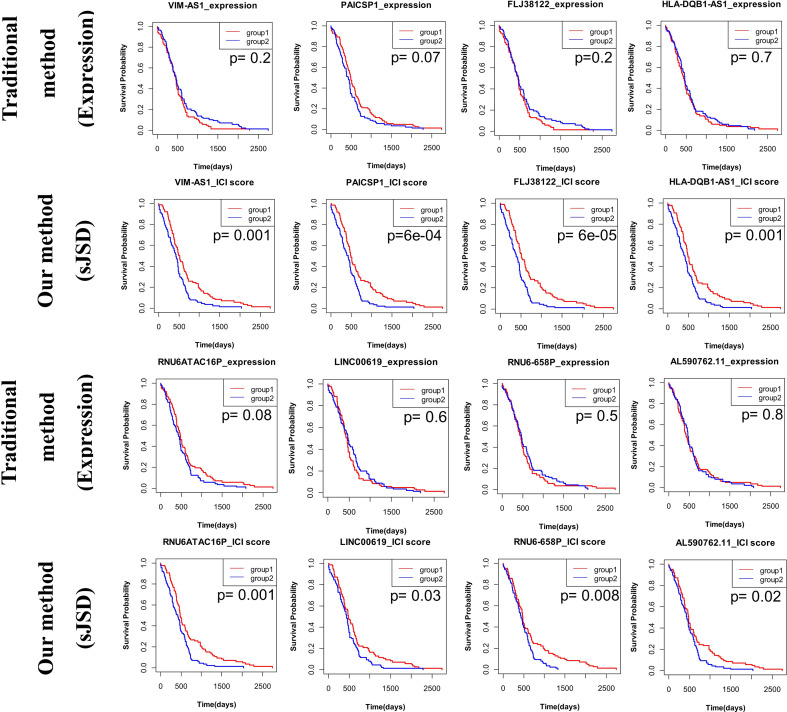
The prognosis analysis based on ‘dark genes’ of PAAD. The prognosis of these “dark genes” respectively based on gene expression and ICI score by dividing the samples into two groups based on the median of genes expression or ICI score, in which Group 1 is a group with higher values and Group 2 is a group with lower values. Eight genes VIM-AS1, FLJ38122, PAICSP1, HLA-DQB1-AS1, RNU6ATAC16P, RNU6-658P, LINC00619, and AL590762.11, none of which is differentially expressed in stage IB (the critical stage before distant metastasis), perform well in CESC prognosis. All of these genes are ‘positive dark genes’.

For PAAD, **Figure 11** shows the survival analysis of VIM-AS1, FLJ38122, PAICSP1, and other five genes results with P-values < 0.05. Based on some non-differential genes in sJSD signal biomarker groups of PAAD. A higher ICI score in “positive dark genes”, i.e., VIM-AS1, FLJ38122, PAICSP1, HLA-DQB1-AS1, RNU6ATAC16P, RNU6-658P, LINC00619, and AL590762.11, is significantly related to a good prognosis (**Table 4**). This validated the effectiveness of the ‘dark gene’ for the development of PAAD. Therefore, The ICI of some critical genes could be an effective indicator of genetic importance and supplement for patients’ prognosis.

## Discussion

Exploring the warning signals of sudden deterioration is essential for identifying the most complex diseases. The lack of samples makes it difficult to detect the critical state prior to the appearance of obvious symptoms. Identifying the early warning signal of critical state for complex diseases based on an individual’s single-sample data is a crucial problem in current research. In the study, we proposed a sJSD method based on a single sample to quantify the information loss when the reference distribution is used to fit the disturbance distribution. This method converts the molecular expression data into a cumulative area of the molecules’ probability distribution. Based on the sJSD algorithm, the ICI is constructed to quantify the differences between reference distribution and disturbed distribution so as to detect complex diseases’ critical state and reveal “dark genes” in the leading network during disease progression.

In this study, JSD is used to detect the critical state by quantifying the difference between reference samples and case samples during disease progression. The case samples are from samples of an individual at multiple time points or stages. Before the sJSD value reaches the peak, it has experienced a period of continuous increase, which corresponds to the process from normal state to critical state of complex disease. The peak in the temporal signals reflects not only the change of the score at one time point or stage, but also the cumulative effect of its phased increase. It is consistent with the progression of the disease. Therefore, the peak in the temporal signals is of great significance in detecting the critical state after a period of potential deterioration. There may be multi-stage deteriorations or multiple tipping points during a cascade deterioration process, which can be also detected by our method. However, we mainly focus on the earliest tipping points in actual clinical applications.

The sJSD method has been applied to five real datasets. In the influenza infection dataset GSE30550, the method successfully identified warning signals of critical transition in nine symptomatic individuals. The calculated results are consistent with the experimental results, which indicate that the sJSD algorithm can effectively identify the samples’ critical state and accurately detect the early warning signals of an individual infected by the influenza virus. Especially, the obvious change of ICI score indicates the critical state (6^th^ hour) of prostate cancer before distant metastasis in the prostate cell line, the critical state (Stage II) of BLCA before symptoms of hydronephrosis or tumor compression, the critical state (Stage IIB) of CESC before cancer begins to infiltrate the cervical tissue, the critical state (Stage IB) of PAAD before lymph node metastasis. The critical states of cancers were verified by prognostic analysis. If patients are diagnosed before the pre-disease state and get appropriate treatment, they will have a better prognostic effect. Furthermore, prognostic analysis of other stages proves no other critical state, indicating that the detected critical stage is precise and closely related to prognosis. The sJSD signal biomarkers and the high-frequency genes in the sJSD signal biomarker group are closely related to disease development’s biological processes and signal pathways.

The sJSD method has four advantages. Firstly, this method mainly studies the dynamic changes of biomolecules structure based on the distribution difference of biomolecules from the network level. Compared with the direct use of molecule expressions, it can effectively remove or reduce errors caused by inaccurate measurement and individual differences of patients. Secondly, the method can detect critical transitions based on individual’s single-sample data, which can be applied to individualized medical diagnosis and individual specificity analysis in the future. Thirdly, the method can help to reveal some “dark genes” that without differential expressions but are sensitive to ICI score, especially, the prognosis based on the ICI score performs better than expressions of these genes. Finally, this method is model-free and does not require large quantities of data for model training and feature screening. Only a index need to be constructed based on JSD theory, i.e., the ICI. It is, therefore, of great potential in personalized pre-disease diagnosis.

## Conclusion

We propose a sJSD method based on single-sample information that can detect early warning signals of pre-disease state before disease deterioration. The method is model-free and has high sensitivity, which can be applied to individuals’ specific diagnosis and the research of some targeted drugs. Besides, identifying sJSD signal biomarkers is also of great significance for exploring disease progression’s potential molecular mechanism, discovering new network biomarkers and ‘positive or negative dark genes’.

## Data Availability Statement

The original contributions presented in the study are included in the article/**Supplementary Material**. Further inquiries can be directed to the corresponding authors.

## Author Contributions

JY and PL developed the methodology. JY executed the experiments. RG and YL helped the experiments and provided technical support. JY and PL wrote and revised the manuscript. LC and PL supervised the work and critically reviewed the paper. All authors contributed to the article and approved the submitted version.

## Funding

This work was funded by the National Key R&D Program of China (No. 2017YFA0505500), National Natural Science Foundation of China (Nos. 31771476, 31930022, 61673008), Shanghai Municipal Science and Technology Major Project (No. 2017SHZDZX01) and the Young Backbone Teacher Funding Scheme of Henan (No.2019GGJS079).

## Conflict of Interest

The authors declare that the research was conducted in the absence of any commercial or financial relationships that could be construed as a potential conflict of interest.
