# Supplementary material for: Identifying Critical States of Complex Diseases by Single-Sample Jensen-Shannon Divergence
Source: Front Oncol. 2021 Jun 4;11:684781. doi: 10.3389/fonc.2021.684781 (PMC8212786; doi:10.3389/fonc.2021.684781)
Supplement: Supplementary file 1 [file DataSheet_1.docx]

Supplementary Material：Identifying critical state by single-sample Jensen-Shannon divergence

**Contents**

**A. Validating the identified critical state by The Kaplan-Meier (log-rank) survival analysis** S1

A1. Validating the identified critical state for BLCA S2

A2. Validating the identified critical state for CESC S3

A3. Validating the identified critical state for PAAD S4

**B. The functional analysis for CESC and PAAD** S5

B1. The functional analysis for BLCA S5

B2. The functional analysis for CESC S6

B3. The functional analysis for PAAD S7

**C.** **The sJSD-specific ‘dark genes’ for BLCA, CESC and PAAD**  S8

C1. The sJSD-specific ‘dark genes’ for BLCA S9

C2. The sJSD-specific ‘dark genes’ for CESC S10

# *A. Validating the identified critical state by The Kaplan-Meier (log-rank) survival analysis*

To validate the identified critical state, the prognostic analysis based on before and after unidentified state were presented and compared through Kaplan-Meier (log-rank) survival analysis. For instance, the following steps were executed to verify a identified critical state of cancer, firstly, detect a critical state of a tumor disease by sJSD methods. Secondly, perform prognostic analysis on the samples before and after identified critical state. Thirdly, carry out prognostic analysis on the samples before and after unidentified other states. Finally, compare the prognostic differences before and after identified critical state and unidentified states. Specifically, there is normally a longer survival time before the identified critical state, than the survival time after the identified critical state. However, there was no significant difference in prognostic analysis before and after the remaining states.

**Table S1 Number of samples within each stage in tumor disease dataset from TCGA.**

# *A1. Validating the identified critical state for BLCA*

**
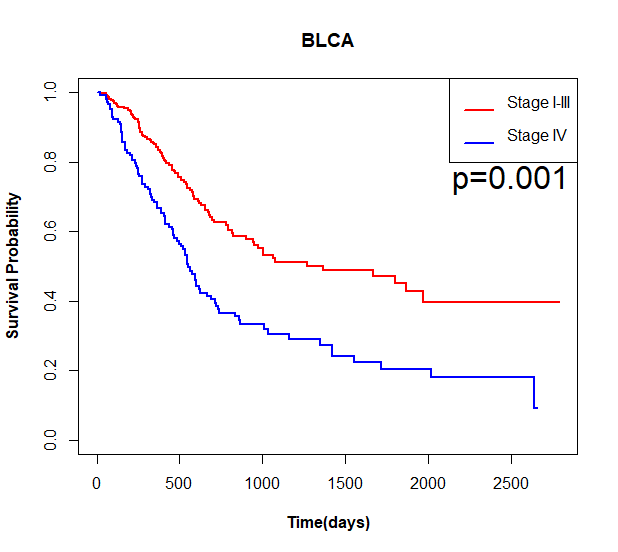

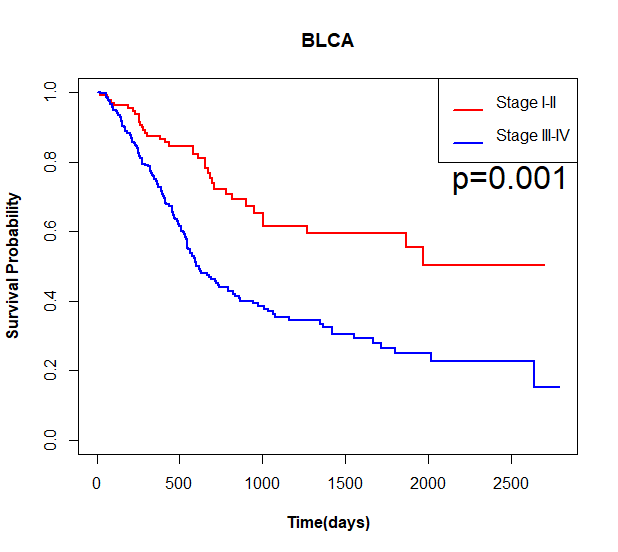

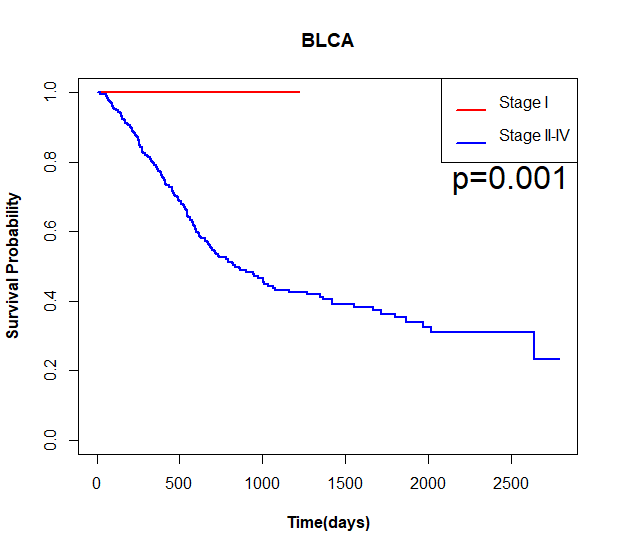
**

**A B C**

**Figure S1: Comparison of survival time for BLCA before and after every state (including the identified critical state, i.e., Stage II, and unidentified state). (A)** Stage I of BLCA vs the stages after Stage I (II-IV) of BLCA in survival analysis. **(B)** The stages in before-transition period (IA-IIA) of BLCA vs the stages in after-transition period (III-IV) of BLCA in survival analysis. **(C)** The Stage before stage III (I-III) of BLCA vs the stages after Stage III (IV) of BLCA in survival analysis.

# *A2. Validating the identified critical state for CESC*

**
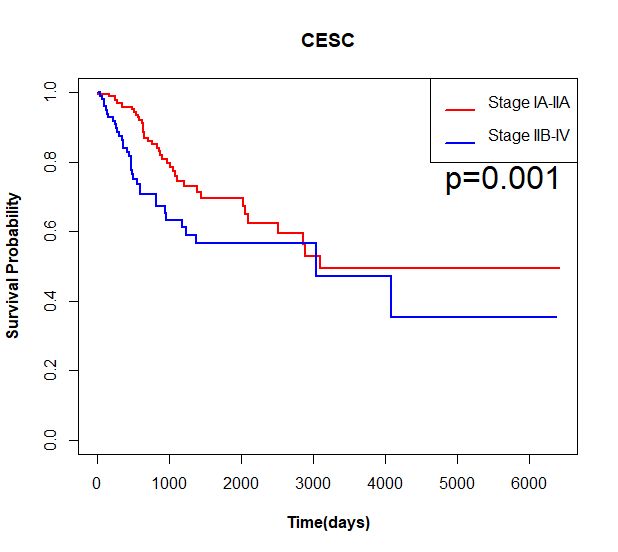

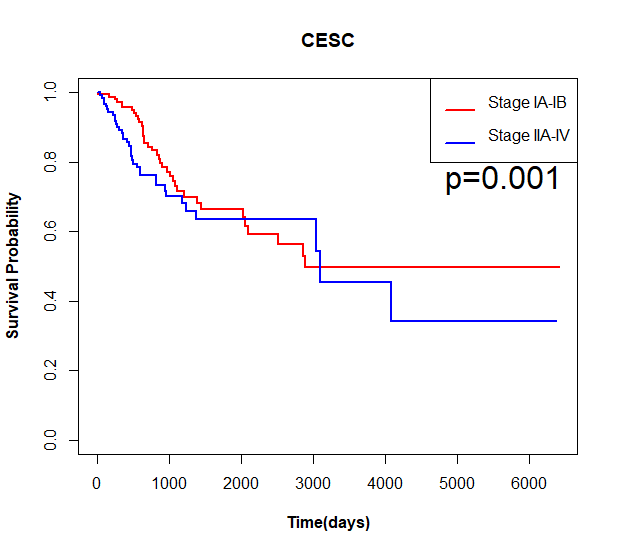

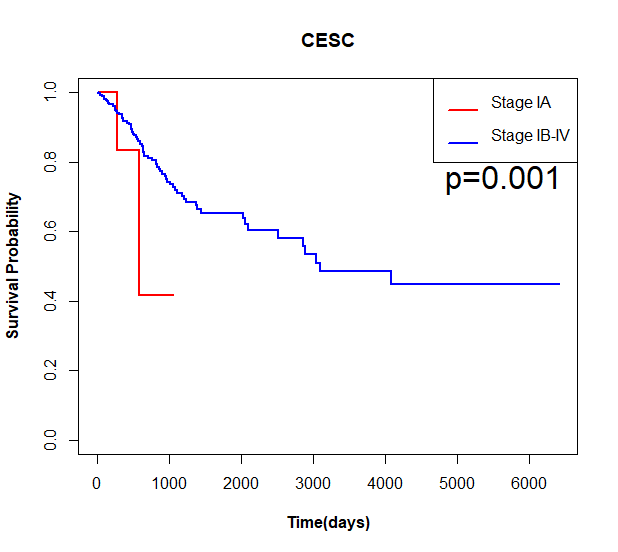
**

**
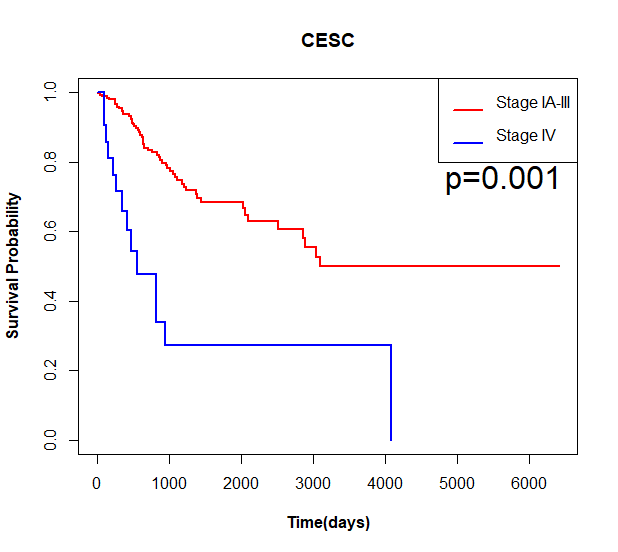

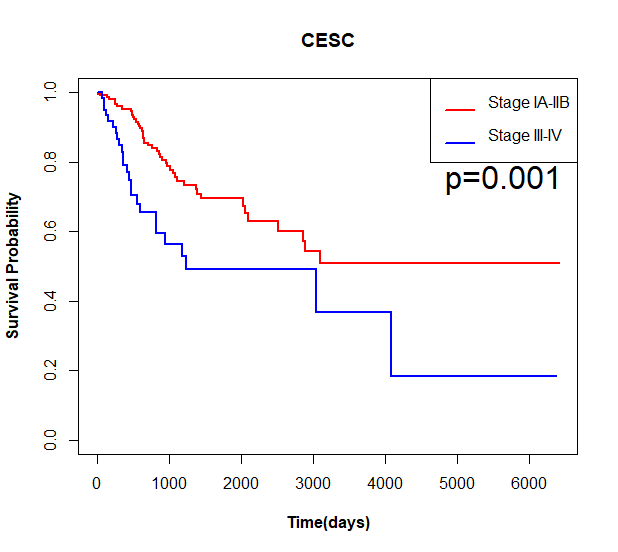
A B C**

**D E**

**Figure S2: Comparison of survival time for CESC before and after every state (including the identified critical state, i.e., Stage IIB, and unidentified state). (A)** Stage IA of CESC vs the stages after Stage IA (IB-IV) of CESC in survival analysis. (**B)** The Stage before stage IB (IA-IB) of CESC vs the stages after Stage IB (IIA-IV) of CESC in survival analysis. (**C)** The Stage before stage IIA (IA-IIA) of CESC vs the stages after Stage IIA (IIB-IV) of CESC in survival analysis. (**D)** The stages in before-transition period (IA-IIB) of CESC vs the stages in after-transition period (III-IV) of CESC in survival analysis. (**E)** The Stage before stage III (IA-III) of CESC vs the stages after Stage III (IV) of CESC in survival analysis.

# *A3. Validating the identified critical state for PAAD*


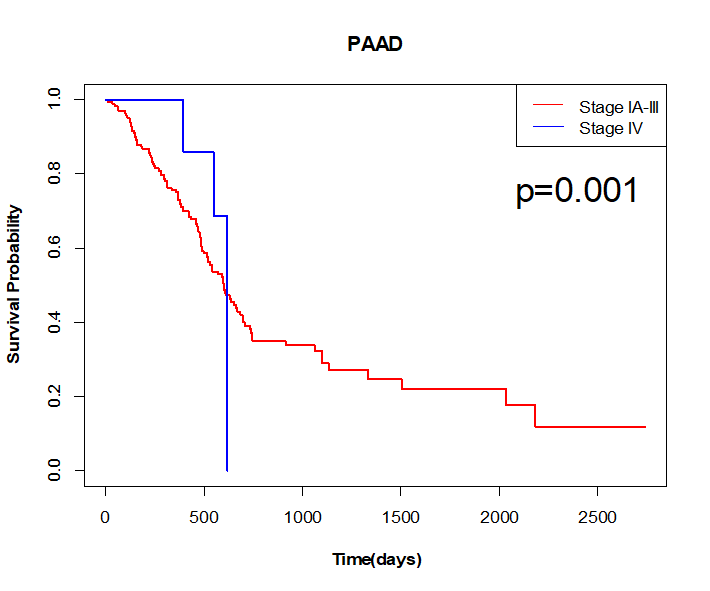
**A B**

**C D**

**Figure S3: Comparison of survival time for PAAD before and after every state (including the identified critical state, i.e., Stage IB, and unidentified state). A** Stage IA of PAAD vs the stages after Stage IA (IB-IV) of PAAD in survival analysis. **B** The stages in before-transition period (IA-IB) of PAAD vs the stages in after-transition period (II-IV) of PAAD in survival analysis. **C** The Stage before stage II (IA-II) of PAAD vs the stages after Stage II (III-IV) of PAAD in survival analysis. **D** The Stage before stage III (IA-III) of PAAD vs the stages after Stage III (IV) of PAAD in survival analysis.

# *B. The functional analysis for BLCA, CESC and PAAD*

# *B1. The functional analysis for BLCA*

**Table S2. The functional enrichment of high-frequency “sJSD signal markers” in the critical stage samples for BLCA**

| Gene Ontology Consortium |  |  | KEGG |  |
| --- | --- | --- | --- | --- |
| enriched biological process | enriched p value |  | enriched biological process | enriched p value |
| adenylate cyclase-modulating G-protein coupled receptor signaling pathway (GO:0007188) | 0.013084 |  | Adrenergic signaling in cardiomyocytes | 0.008837 |
| apoptotic process involved in heart morphogenesis  (GO:0003278) | 0.014048 |  | Estrogen signaling pathway | 0.020762 |
| collagen fibril organization (GO:0030199) | 0.01447 |  | cAMP signaling pathway | 0.029398 |
| extracellular space (GO:0005615) | 0.002584 |  | Ovarian steroidogenesis | 0.034167 |
| glutathione derivative biosynthetic process (GO:1901687) | 0.004749 |  | AMPK signaling pathway | 0.036328 |

# *B2. The functional analysis for CESC*

**Table S3. The high frequency genes in 43 “sJSD signal markers” groups in the critical stage (stage IIB) for CESC**

| Gene Id | Gene name | Frequency | Location | Family | Relation with cancer progression |
| --- | --- | --- | --- | --- | --- |
| ZIM2 | Zinc Finger Imprinted 2 | 29 | Nucleus | transcriptional regulation/Protein Code | Zim2 is found to be a new mutant gene during  Cancer[42]. |
| PCDH11X | Protocadherin 11 X-Linked | 24 | Plasma membrane | Phosphatase | The cytodomain of PCDH11X has been shown to interact with β‐catenin,inducing the Wnt signaling pathway in cultured cancer cells[43]. |
| AGBL4 | ATP/GTP Binding Protein Like 4 | 23 | Cytoplasm | peptidase | AGBL4 is identified as a specific gene for cancer[44]. |
| DACH2 | Dachshund Homolog 2 | 23 | Nucleus | transcription Factor | DACH2 is an independent prognostic marker that can be used at initial diagnosis of cancer(UCB) to identify patients who have a high potential to develop metastasis[45]. |
| EPHA5 | EPH Receptor A5 | 20 | Endoplasmic reticulum | Kinase | EphA5 is abnormally expressed in numerous malignant tumors and may be involved in the radiosensitivity of cancer[46]. |
| LINC01016 | Long Intergenic Non-Protein Coding RNA 1016 | 20 | Nucleus | other | miRNAs could inhibit LINC01016 transcription, forming two reciprocal repression cycles, which influenced the biological behavior of cancer cells[47]. |
| PGM5-AS1 | PGM5 Antisense RNA 1 | 20 | Golgi apparatus | RNA | PGM5-AS1 was downregulated in human colorectal cancer tissues and cells[48]. |

**Table S4. The functional enrichment of high-frequency “sJSD signal markers” in the critical stage samples for CESC**

| Gene Ontology Consortium |  |  | KEGG |  | |
| --- | --- | --- | --- | --- | --- |
| enriched biological process | enriched p value |  | enriched biological process | | enriched p value |
| axon guidance (GO:0007411) | 0.012341195 |  | Dilated cardiomyopathy (DCM) | | 0.0185 |
| enzyme binding (GO:0019899) | 0.043411883 |  | Hypertrophic cardiomyopathy (HCM) | | 0.0218 |
| focal adhesion (GO:0005925) | 0.007356628 |  | Insulin secretion | | 0.0234 |
| homeostasis of number of cells within a tissue (GO:0048873) | 0.030649364 |  | cGMP-PKG signaling pathway | | 0.0365 |
| identical protein binding (GO:0042802) | 0.037175191 |  | Cholinergic synapse | | 0.0365 |
| Mitochondrion (GO:0005739) | 0.04541451 |  | Protein digestion and absorption | | 0.0365 |

# *B3. The functional analysis for PAAD*

**Table S5. The high frequency genes in 15 “sJSD signal markers” groups in the critical stage (stage IB) for PAAD**

| Gene Id | Gene name | Frequency | Location | Family | Relation with cancer progression |
| --- | --- | --- | --- | --- | --- |
| HNRNPCL1 | Heterogeneous Nuclear Ribonucleoprotein C Like 1 | 11 | Nucleus | Ribonucleoprotein | HNRNPCL1 is indicative for an increased probability to suffer from pancreatic cancer[52]. |
| LINC00682 | Long Intergenic Non-Protein Coding RNA 682 | 11 | Cytoplasm | RNA Gene | LINC00682 methylation signature was significantly associated with short recurrence‐free survival in patients[53]. |
| LINC01180 | Long Intergenic Non-Protein Coding RNA 1180 | 11 | Cytoplasm | RNA Gene | LINC01180 has a role in physiological and pathological processes, including cancer[54]. |
| MORC1 | MORC Family CW-Type Zinc Finger 1 | 10 | Nucleus | Transcriptional regulation | MORC was expressed in 36% in ten CT genes (The Cancer-testis (CT) antigens are expressed in many malignant tumors)[55]. |
| MIR656 | MicroRNA 656 | 9 | Cytoplasm | RNA Gene | MiR‐656 influence the proliferation and migration of cancer-related cells[56]. |
| LINC00906 | Long Intergenic Non-Protein Coding RNA 906 | 9 | Cytoplasm | RNA Gene | LINC00906 involved in cellular differentiation and proliferation as post-transcriptional regulators of splicing or as molecular decoys for miRNA[57]. |
| RGPD6 | RANBP2 Like AndGRIP Domain Containing 6 | 8 | Cytoplasm | transcription regulator | RGPD6 was the most mutated gene in tumours and mostly enriched in cellular and metabolic processes[58]. |
| MIR1250 | MicroRNA 1250 | 8 | extracellular region | Other | MiR-1250 is located in 17 q25.3, whose genetic phenotype is often closely related to malignant biological behavior such as vascular invasion and distant metastasis of tumors[59]. |

**Table S6. The functional enrichment of high-frequency “sJSD signal markers” in the critical stage samples for PAAD**

| Gene Ontology Consortium |  |  | KEGG |  |
| --- | --- | --- | --- | --- |
| enriched biological process | enriched p value |  | enriched biological process | enriched p value |
| immune system process(GO:0002376) | 1.62E-22 |  | Allograft rejection | 4.58E-05 |
| regulation of immune system process(GO:0002682) | 1.06E-20 |  | Cytokine-cytokine receptor interaction | 4.24E-06 |
| positive regulation of immune system process(GO:0002684) | 1.13E-19 |  | Graft-versus-host disease | 5.35E-05 |
| cell surface receptor signaling pathway(GO:0007166) | 1.63E-16 |  | Herpes simplex infection | 0.000003 |
| signal transduction(GO:0007165) | 3.61E-15 |  | Inflammatory bowel disease (IBD) | 7.78E-06 |

# *C. The sJSD-specific ‘dark genes’ for BLCA, CESC and PAAD*

Based on the sJSD algorithm proposed in Method section of the main text (the 5 steps of calculating sJSD), the ’dark genes’ is identified in the following ways.

**Step1.** Preparing some control samples and one single case sample, in which the n control samples are reference, and the single case sample is to-be-check case sample.

**Step2.** Calculate the ICI score for every genes based on the sJSD algorithm.

**Step3.** To verify the role of the sJSD signal markers in in cancer treatment or progression, the prognosis analysis of every gene was carried out based on its ICI score and gene expression data.

**Step4.** Some of the sJSD signal markers are viewed as ‘dark genes’, if these genes are non-differential, and couldn’t be detected as critical markers in traditional methods.

**Step5.** A gene is regarded as a “positive dark gene”, if it performs well in tumor prognosis analysis, i.e., a higher ICI score in the gene brings about a longer survival time, conversely, a gene is considered as a “negative dark gene”, if it performs poor in tumor prognosis analysis, i.e., a higher ICI score in the gene brings about a shorter survival time.

#
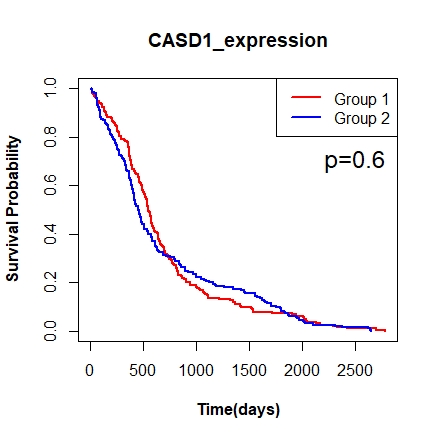

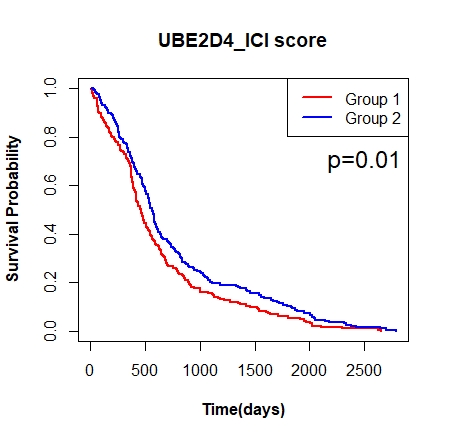

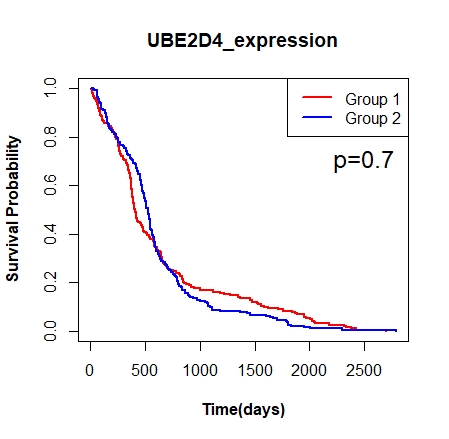

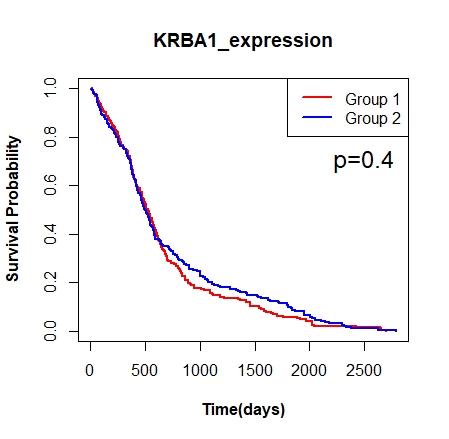

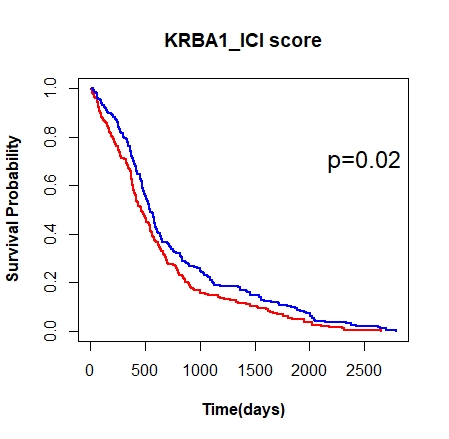

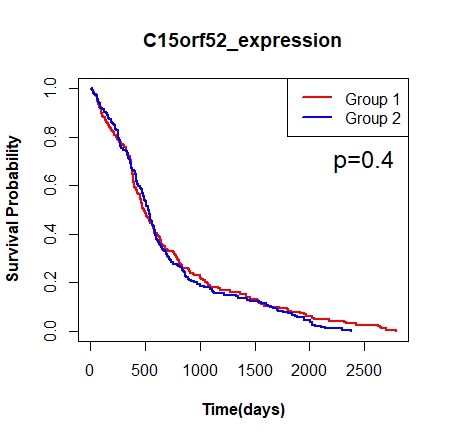

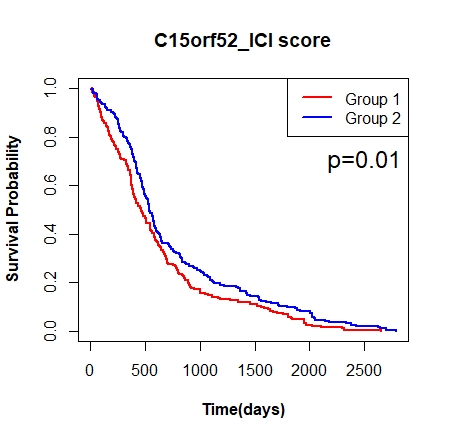

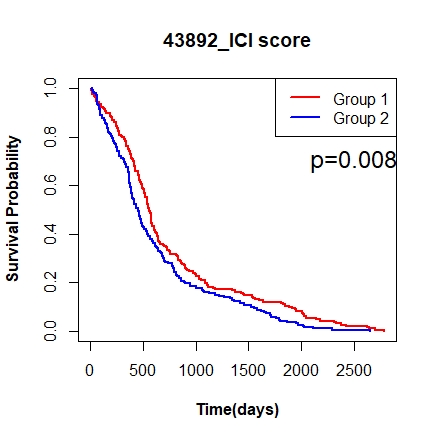

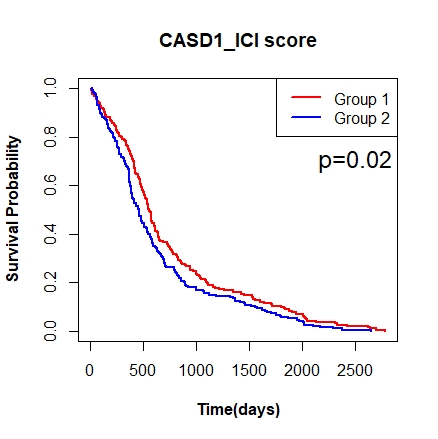

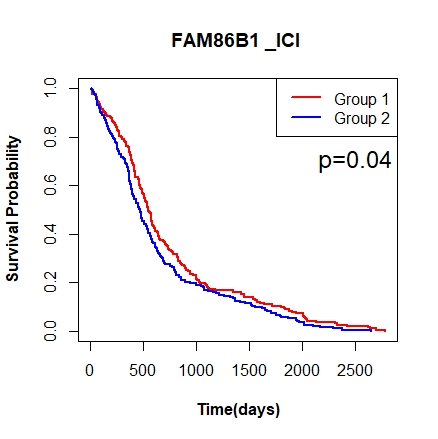

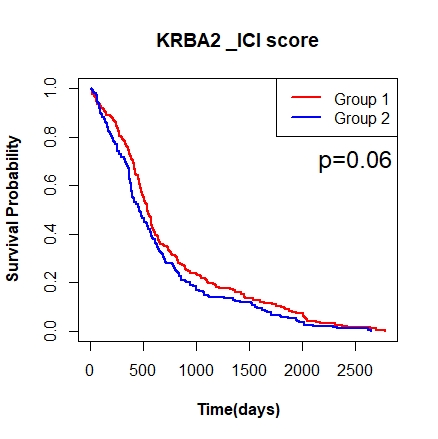

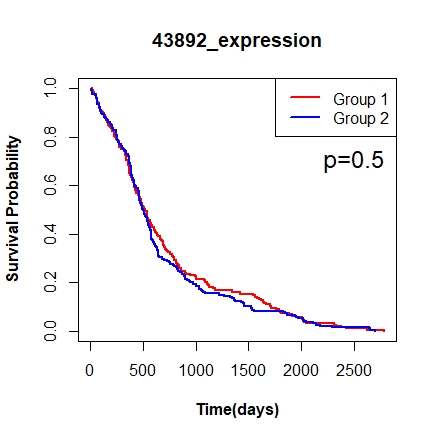

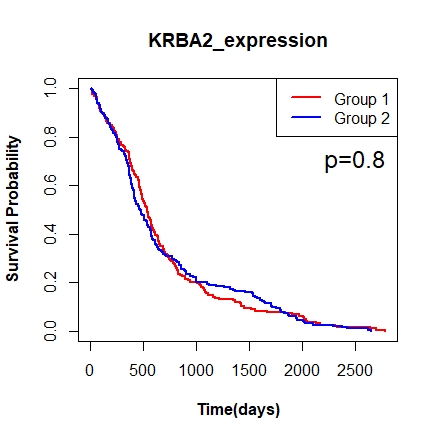

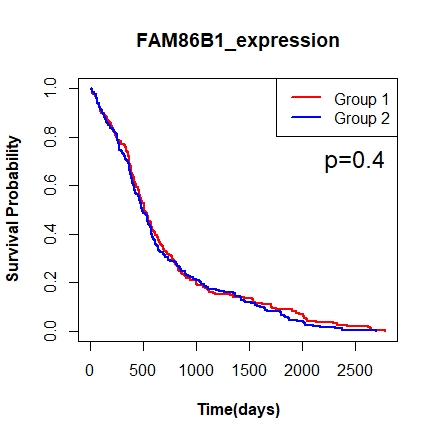
*C1. The sJSD-specific ‘dark genes’ for BLCA*

**Our method**

**(sJSD)**

**Traditional**

**method**

**(Expression)**

**Our method**

**(sJSD)**

**Traditional**

**method**

**(Expression)**

**Figure S4: The prognosis analysis based on ‘dark genes’ of BLCA**

#
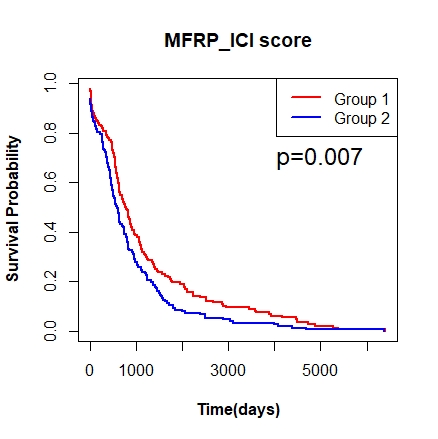

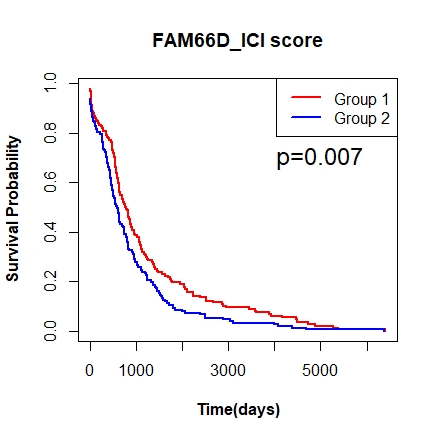

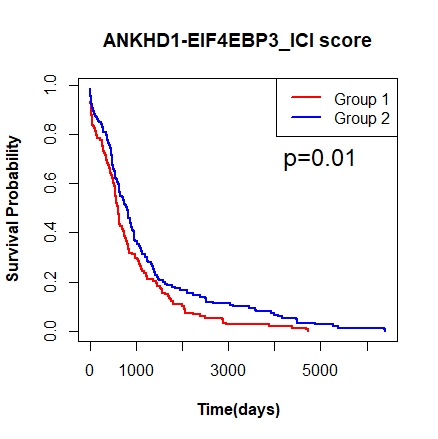

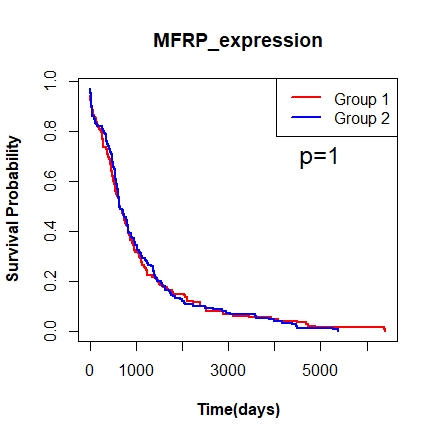

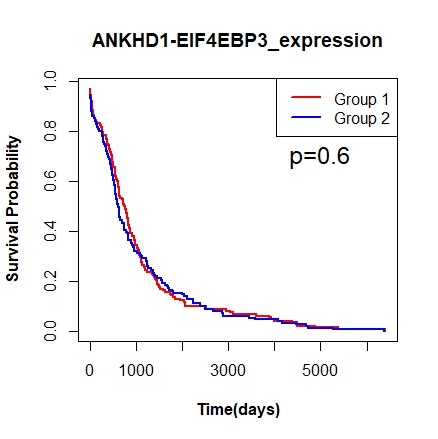

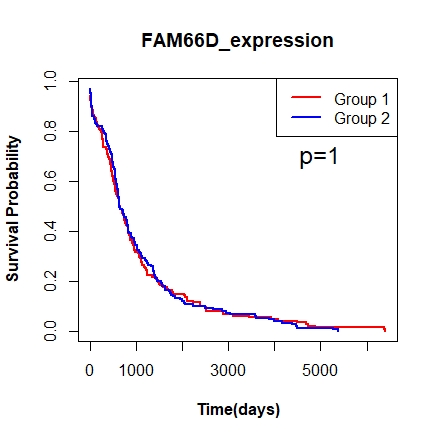

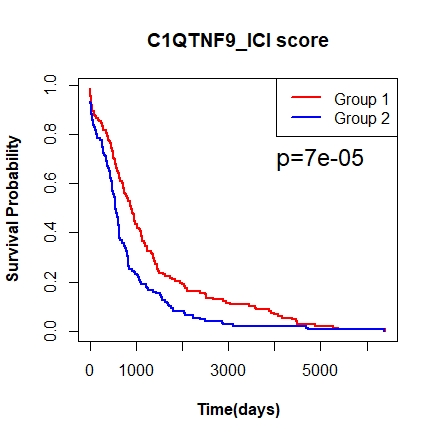

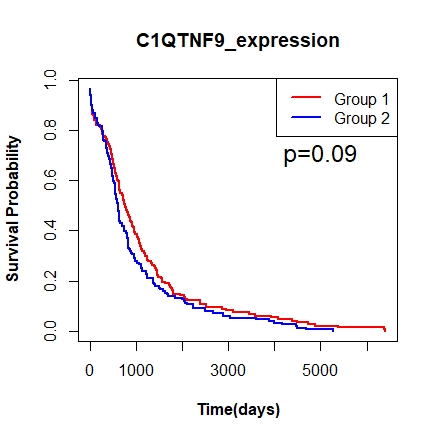

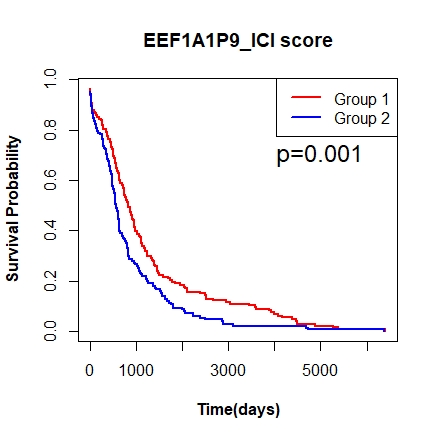

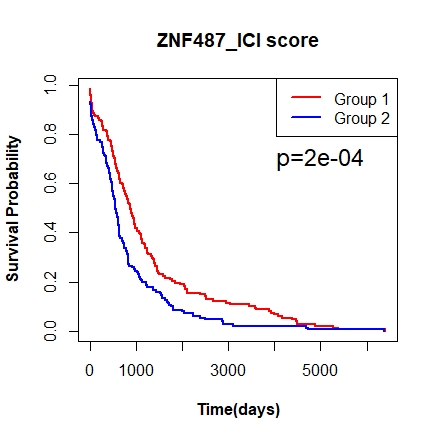

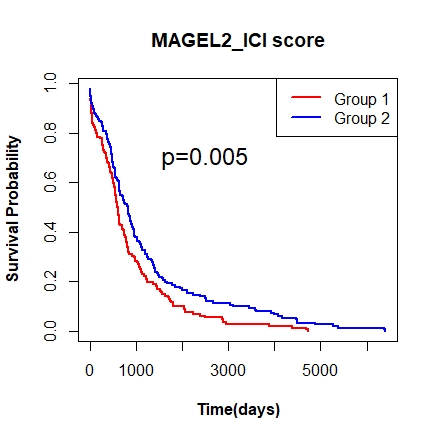

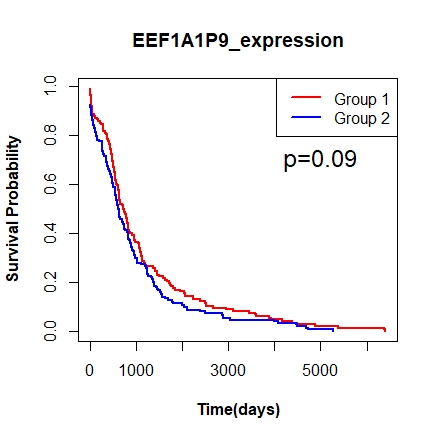

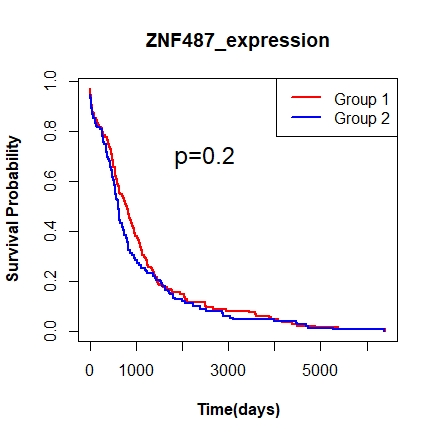

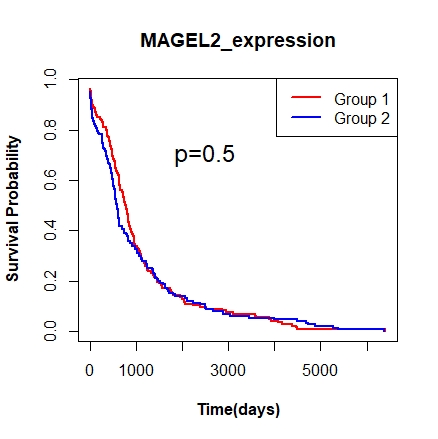
*C2. The sJSD-specific ‘dark genes’ for CESC*

**Our method**

**(sJSD)**

**Traditional**

**method**

**(Expression)**

**Our method**

**(sJSD)**

**Traditional**

**method**

**(Expression)**

**Figure S5: The prognosis analysis based on ‘dark genes’ of CESC**
