# Supplementary figures and images for: Identifying Critical States of Complex Diseases by Single-Sample Jensen-Shannon Divergence
Source: Front Oncol. 2021 Jun 4;11:684781. doi: 10.3389/fonc.2021.684781 (PMC8212786; doi:10.3389/fonc.2021.684781)

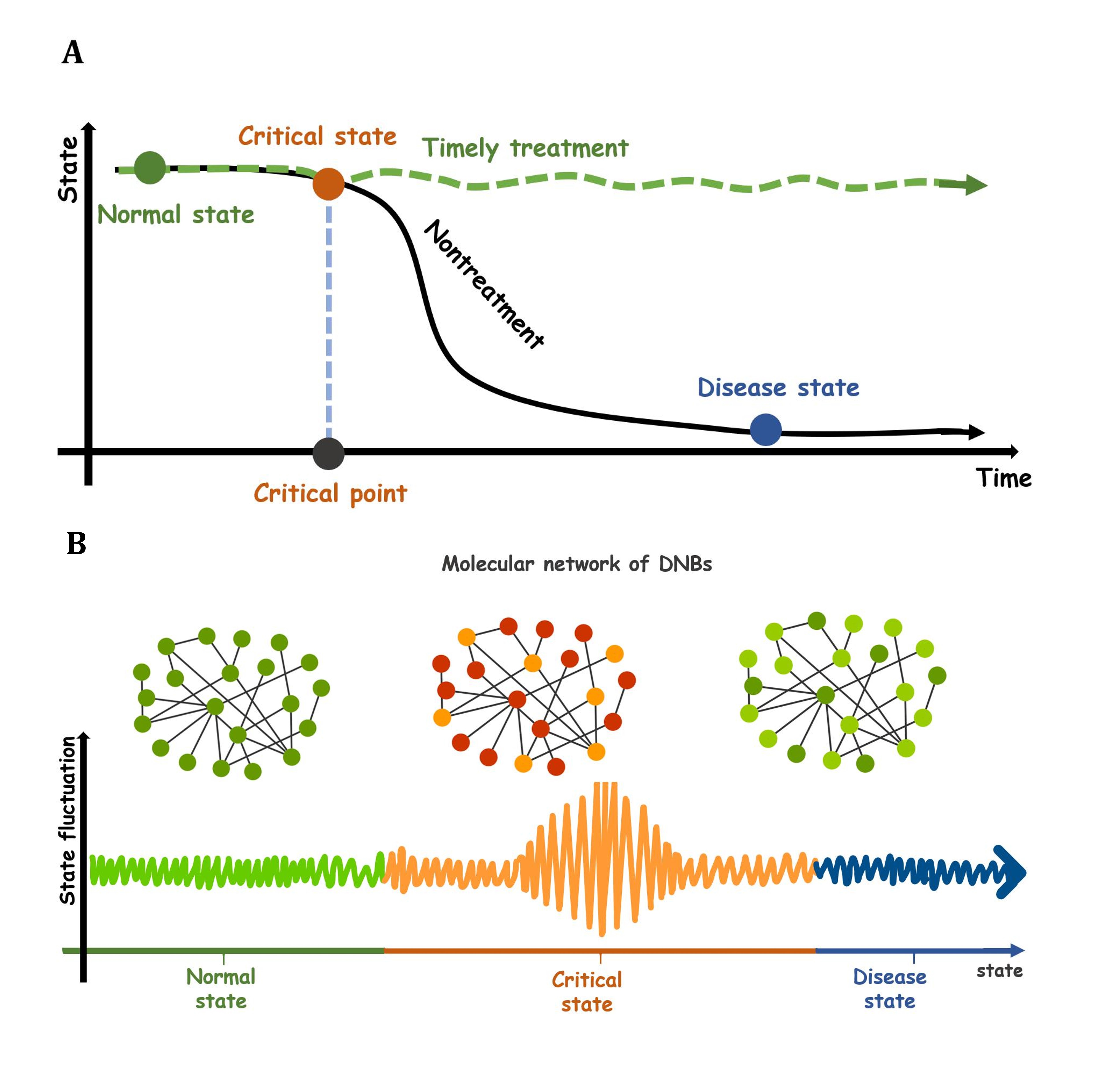

Supplement: Supplementary file 8 [file DataSheet_8.zip › Supplementary Material Presentation/FIG. 1. Dynamic evolution of complex diseases.jpg]

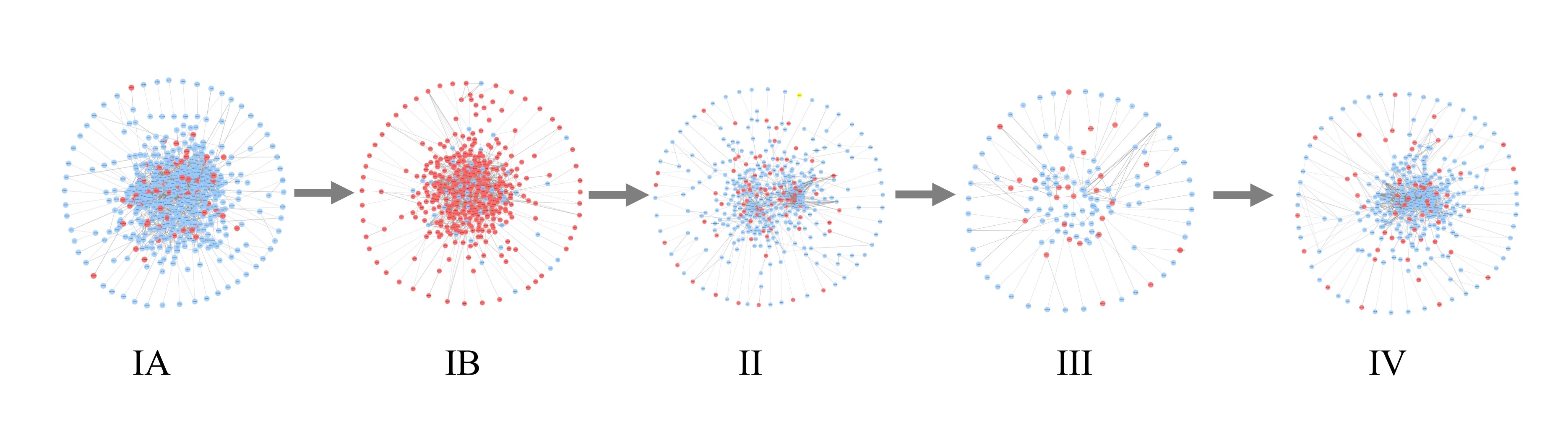

Supplement: Supplementary file 8 [file DataSheet_8.zip › Supplementary Material Presentation/FIG. 10. The dynamic evolution of the sJSD signal markers for PAAD.jpg]

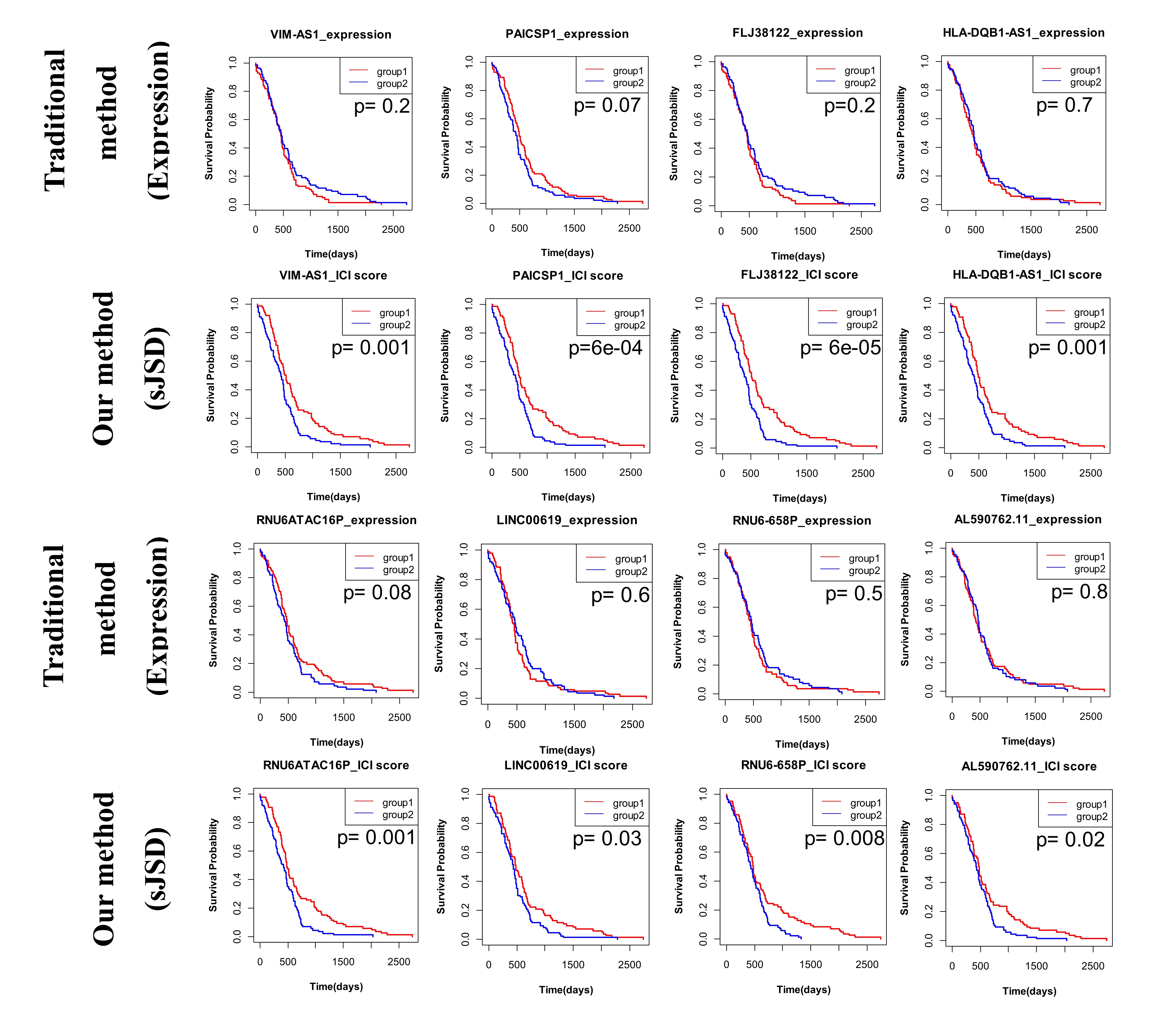

Supplement: Supplementary file 8 [file DataSheet_8.zip › Supplementary Material Presentation/FIG. 11. The prognosis analysis based on ‘dark genes’ of igure 11 The prognosis analysis based on ‘dark genes’ of PAAD.jpg]

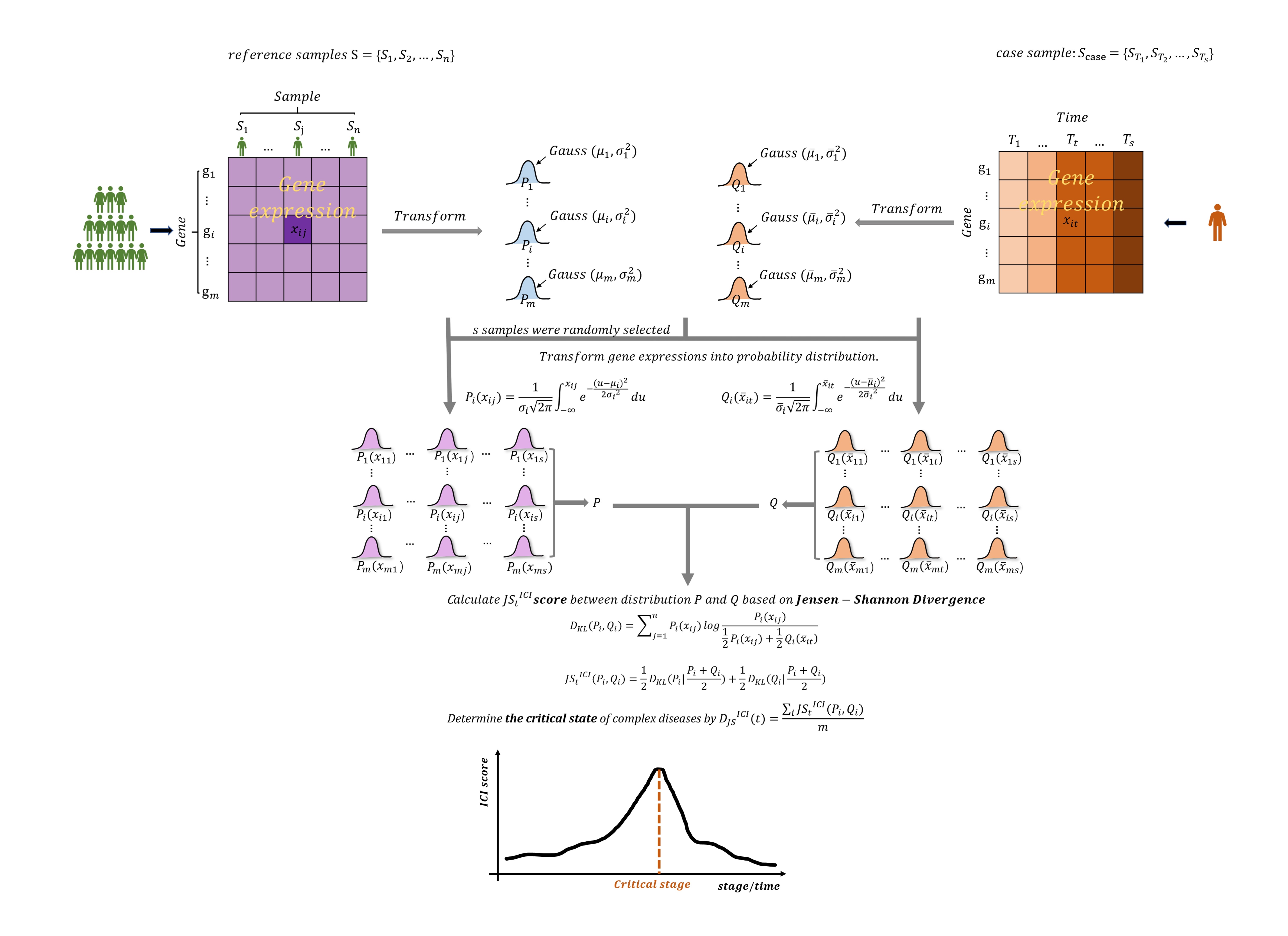

Supplement: Supplementary file 8 [file DataSheet_8.zip › Supplementary Material Presentation/FIG. 2. The outline for detecting early warning signal of pre-disease state based on sJSD.jpg]

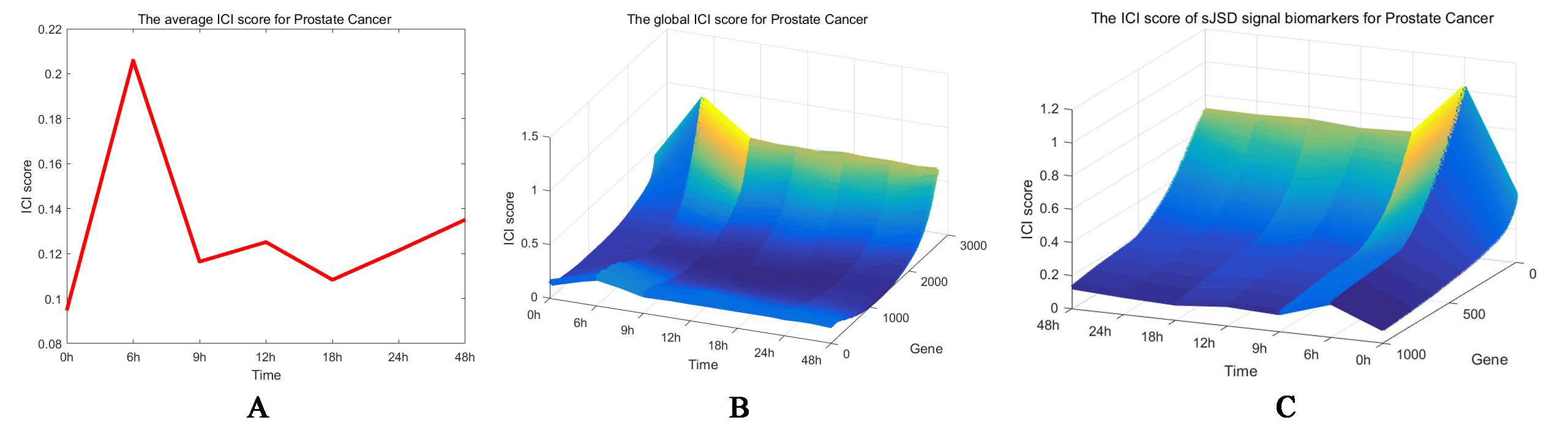

Supplement: Supplementary file 8 [file DataSheet_8.zip › Supplementary Material Presentation/FIG. 3. Application of sJSD method in prostate cancer.jpg]

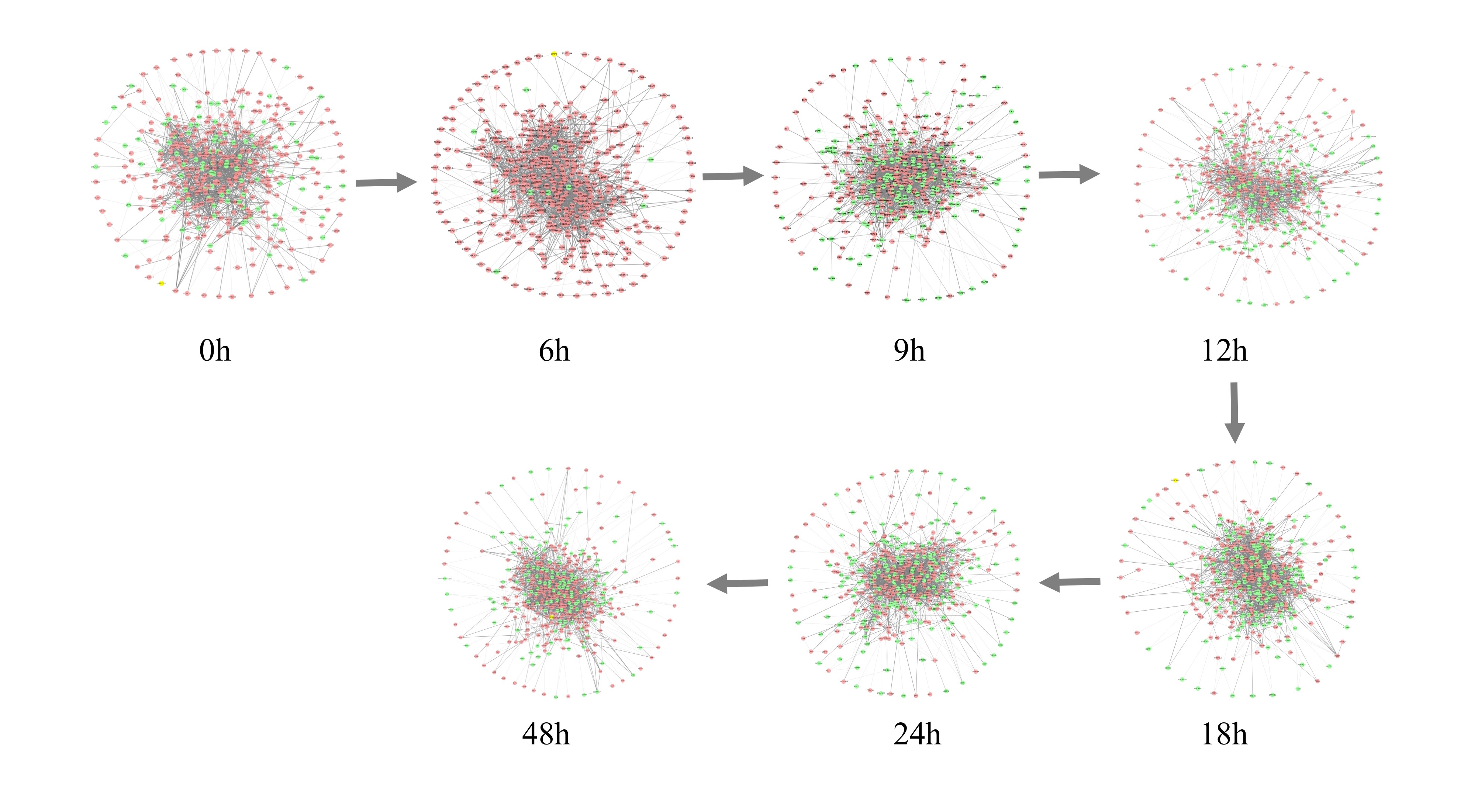

Supplement: Supplementary file 8 [file DataSheet_8.zip › Supplementary Material Presentation/FIG. 4. The dynamic evolution of sJSD signal markers in prostate cancer.jpg]

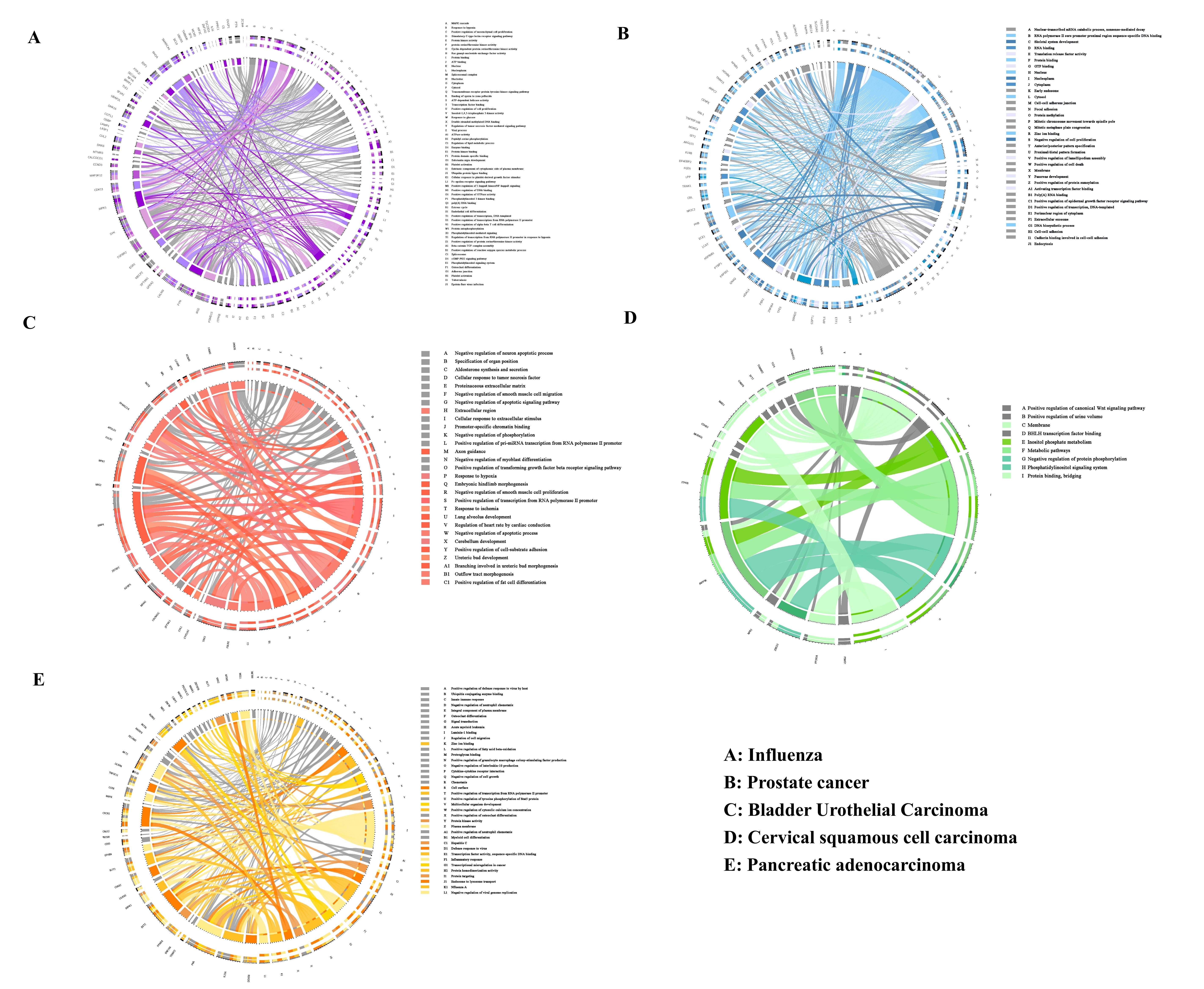

Supplement: Supplementary file 8 [file DataSheet_8.zip › Supplementary Material Presentation/FIG. 5. The sJSD signal biomarkers are involved in important biological processes in five datasets.jpg]

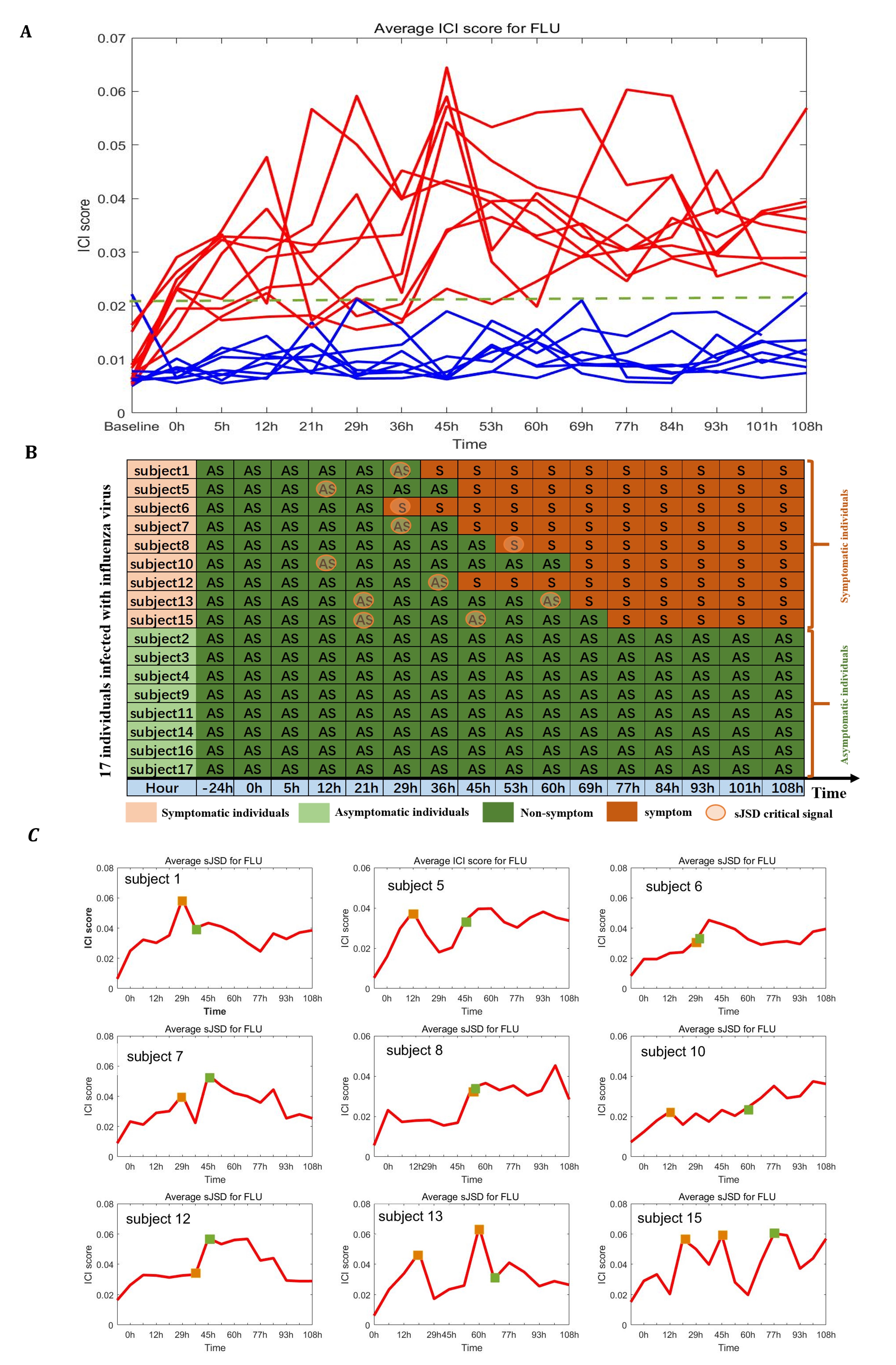

Supplement: Supplementary file 8 [file DataSheet_8.zip › Supplementary Material Presentation/FIG. 6. Identification of critical state of H1N2 influenza infection based on sJSD.jpg]

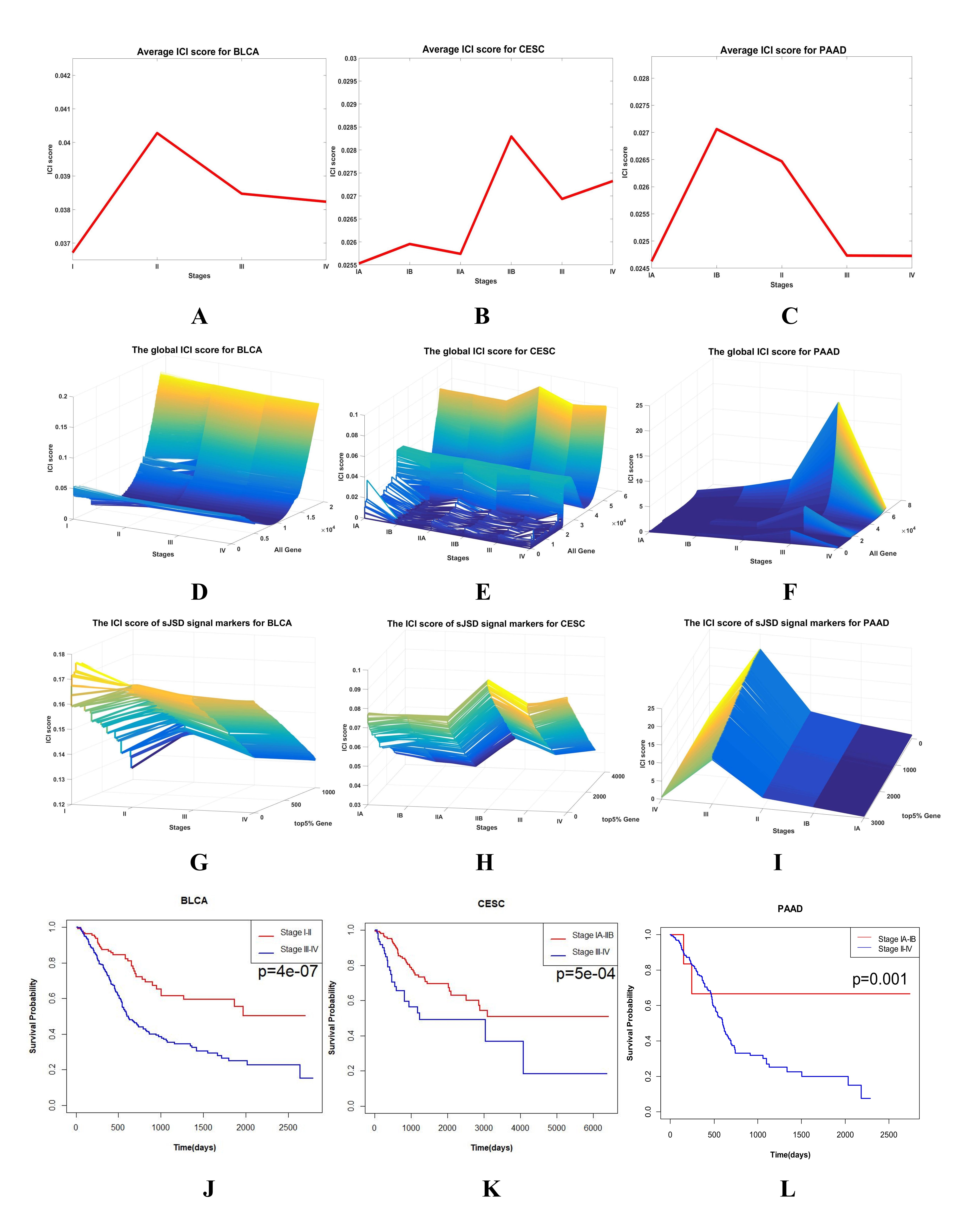

Supplement: Supplementary file 8 [file DataSheet_8.zip › Supplementary Material Presentation/FIG. 7. The application of sJSD in three cancers.jpg]

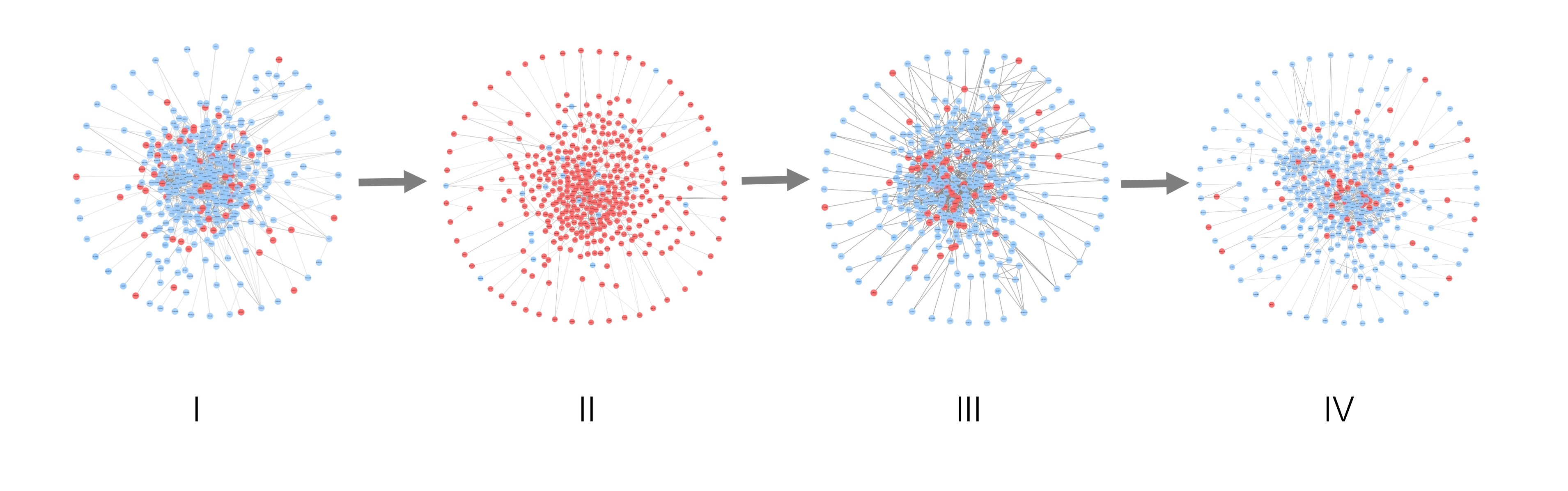

Supplement: Supplementary file 8 [file DataSheet_8.zip › Supplementary Material Presentation/FIG. 8. The dynamic evolution of sJSD signal markers for BLCA.jpg]

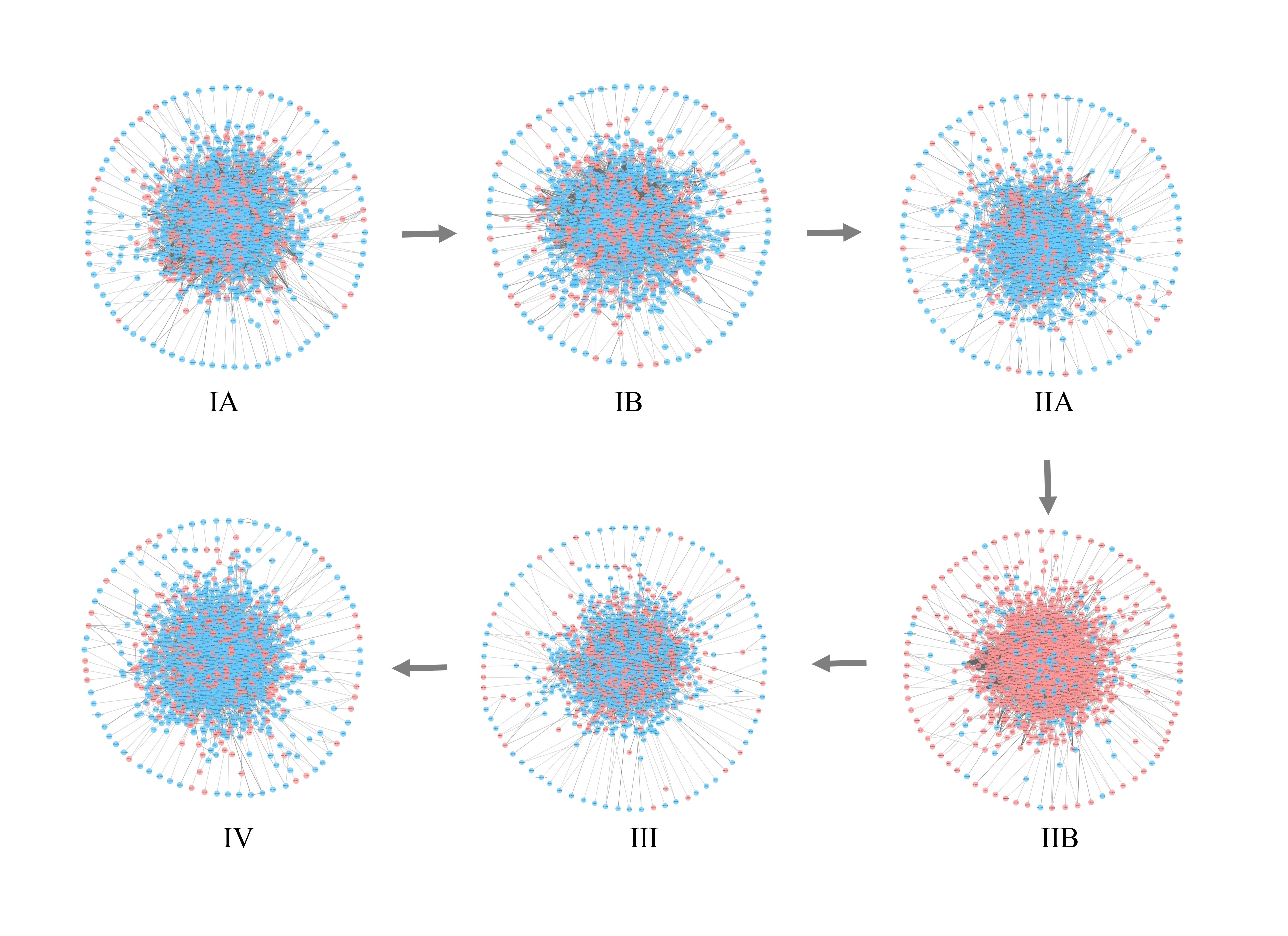

Supplement: Supplementary file 8 [file DataSheet_8.zip › Supplementary Material Presentation/FIG. 9. The dynamic evolution of sJSD signal markers for CESC.jpg]
